# The long non-coding RNA-encoded microprotein MKKS3 drives colorectal cancer chemoresistance

**DOI:** 10.1038/s12276-026-01817-w

**Published:** 2026-09-02

**Authors:** Hui-Fang Hu, Qi Zhou, Jia-Zhen Huang, Jia-Ying Fu, Zhi-Ting Zuo, Qing-Yu He

**Affiliations:** 1 MOE Key Laboratory of Tumor Molecular Biology and State Key Laboratory of Bioactive Molecules and Druggability Assessment, Guangdong Basic Research Center of Excellence for Natural Bioactive Molecules and Discovery of Innovative Drugs, College of Life Science and Technology, Jinan University, Guangzhou, 510632 China; 2 The First Affiliated Hospital of Jinan University, Jinan University, Guangzhou, 510632 China

**Keywords:** Cancer therapeutic resistance, DNA damage response, Diagnostic markers

## Abstract

Although oxaliplatin is widely used in the frontline treatment of colorectal cancer (CRC), CRC recurrence is commonly observed owing to oxaliplatin resistance. The roles of long non-coding RNAs-encoded microproteins in oxaliplatin chemoresistance remain inadequately explored. In this study, a CRISPR/Cas9 library was developed to target previously identified 314 microproteins translated from long non-coding RNAs, followed by functional screening. This strategy identified MKKS3, encoded by NR_072977, as a key regulator of chemoresistance in CRC. A pan-cancer analysis integrating translatomics and proteomics revealed widespread MKKS3 expression across various human cancers. Our experiments demonstrated that MKKS3 promotes chemoresistance in CRC and drives iCAF-to-myCAF conversion. Mechanistically, MKKS3 interacts with TRIM29 to facilitate DNA repair complex assembly and induce STING ubiquitination, thus suppressing IFNβ secretion and further activating fibroblasts. These activated fibroblasts secrete increased amounts of TGFβ, which in turn downregulates STING expression. Additionally, DENR functions as a suppressor of MKKS3 expression and regulates its translation. The natural compound 1-Hydroxybaccatin I inhibits the MKKS3–TRIM29 interaction, and its combination treatment with oxaliplatin enhances therapeutic outcomes of CRC by promoting CD8^+^ T cell activation and tumour infiltration. Moreover, intratumoural injection of AAV8-shMKKS3 with oxaliplatin produces a synergistic anti-tumour effect. Collectively, MKKS3 drives CRC chemoresistance through the TRIM29–STING–IFNβ–TGFβ axis. This study provides novel insights into the mechanism of MKKS3 in regulating chemoresistance and providing a potential therapeutic target for reversing the chemoresistance of CRC.

**Figure:**
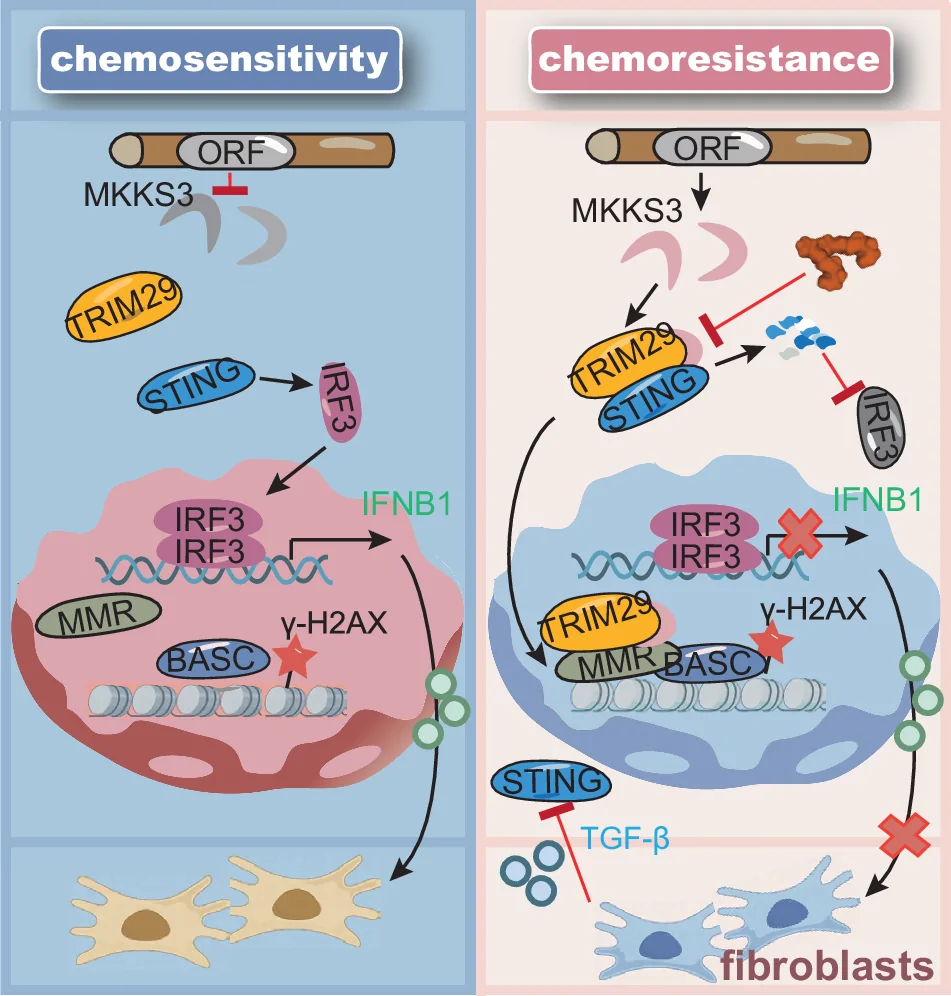

## MKKS3 Drives Colorectal Cancer Chemoresistance via TRIM29

Chemoresistance in colorectal cancer (CRC) remains a significant hurdle, often leading to treatment failure. This study identifies MKKS3, a microprotein encoded by the long non-coding RNA NR_072977, as a key player in CRC chemoresistance. Using CRISPR/Cas9 screening, researchers pinpointed the role of MKKS3 in enhancing oxaliplatin resistance by interacting with TRIM29, facilitating DNA repair and suppressing immune responses. The study employed in vitro and in vivo models to demonstrate that MKKS3 promotes fibroblast activation, creating a feedback loop that further suppresses immune responses and accelerates CRC progression. Notably, 1-Hydroxybaccatin I, a natural compound, disrupts the MKKS3–TRIM29 interaction, enhancing oxaliplatin sensitivity and activating antitumour immunity. These findings suggest that targeting MKKS3 could improve CRC treatment outcomes, offering a promising therapeutic strategy for overcoming chemoresistance and pointing towards future research on MKKS3 inhibitors as potential anti-CRC drugs.

This summary was initially drafted using artificial intelligence, then revised and fact-checked by the author.

## Introduction

Chemoresistance remains a major driver of cancer-related morbidity and mortality, posing a persistent challenge in oncology^1,2^. The mechanisms underlying chemoresistance are multifaceted, including tumour heterogeneity, drug inactivation, evasion of apoptosis, enhanced DNA repair, increased drug efflux, epithelial–mesenchymal transition (EMT), and interactions within the tumour microenvironment (TME)^3,4^. Despite advances in this field, chemotherapy resistance remains the leading cause of treatment failure, leading to poor prognosis, especially in patients with advanced colorectal cancer (CRC). Therefore, current research in CRC is aimed at identifying novel therapeutic targets, enhancing chemotherapy sensitivity and understanding the molecular mechanisms of chemotherapy resistance to develop more effective treatments and improve patient outcomes.

Approximately 2% of the human genome encodes proteins^5^, whereas the remainder has traditionally been classified as non-coding RNAs, including long non-coding RNAs (lncRNAs), primary microRNAs (pri-miRNAs), and circular RNAs (circRNAs)^6^. However, advancements in high-throughput technologies such as ribosome profiling and computational biology have led to a reassessment of this classification. These technologies have uncovered that certain lncRNAs contain small open reading frames (sORFs) capable of encoding functional proteins or peptides^7–9^. The ENCODE database has catalogued about 17,000 lncRNAs, defined as transcripts longer than 200 nucleotides^10^. In our previous work, full-length translating mRNA sequencing and ribosome profiling revealed an unrecognized proteome encoded by 3,300 lncRNAs^11^. Current research increasingly focuses on these microproteins encoded by lncRNAs, particularly their roles in tumorigenesis and their translational implications. For example, the microprotein encoded by the lncRNA CTBP1-DT has been shown to activate dual DNA repair pathways by supporting the DNA damage response^12^, whereas the microprotein SMIM26, encoded by LINC00493, has demonstrated anti-metastatic properties in renal-cell carcinoma by facilitating mitochondrial glutathione import and respiratory efficiency^13^. Also, our previous findings indicated that the protein encoded by LINC00982 inhibits metastasis in CRC by modulating RNA splicing^14^. Research on microproteins, encoded by lncRNAs, related to CRC, is limited and their effects on chemotherapy resistance remain to be clarified.

Cancer-associated fibroblasts (CAFs), activated fibroblastic cells closely associated with tumour cells, constitute a key component of the TME. CAFs play a pivotal role in regulating cancer growth, differentiation and resistance to therapy^15^. For instance, hepatocyte growth factor (HGF) secreted by CAFs has been shown to induce resistance to epidermal growth factor receptor (EGFR)-tyrosine kinase inhibitors in lung cancer, whereas IL-6 produced by CAFs promotes metastasis and chemoresistance via EMT induction^16^. Our ongoing research demonstrated that the abundance of fibroblasts correlates more significantly with patient responses to chemotherapy than the presence of CD8^+^ T cells, natural killer (NK) cells or B cells. Despite this, therapeutic strategies targeting CAFs remain limited, highlighting the need for deeper exploration of their role in tumour chemoresistance. Chemotherapy primarily works by inducing cell death through DNA damage^17^. Recent studies leveraging EMT markers and DNA repair gene panels have enabled stratification of patients with CRC, guiding treatment choices between chemotherapy and immunotherapy to optimize clinical outcomes^18^. A comprehensive review highlights the critical role of PARP inhibitors, particularly in tumours harbouring BRCA1/2 mutations^19^, where the synthetic lethal interaction between PARP inhibition and BRCA1/2 deficiencies exemplifies successful clinical translation^20^. Nonetheless, chemotherapy resistance arises from multiple factors, and the regulatory interactions between fibroblasts and DNA damage repair mechanisms remain poorly understood.

This study conducted an extensive functional screening using a CRISPR/Cas9 library on the 314 lncRNA-encoded proteins identified in our previous research^11^. This led to the identification of MKKS3, a microprotein derived from NR_072977, as a new player in regulating chemoresistance. A series of experiments validated the endogenous expression of MKKS3, and both in vitro and in vivo analyses demonstrated its role in enhancing oxaliplatin resistance in CRC cells. Mechanistically, MKKS3 interacts with TRIM29 to facilitate the assembly of DNA repair complexes and induces the ubiquitination and degradation of STING, thereby inhibiting IFNβ secretion and further activating fibroblasts. These activated fibroblasts secrete elevated levels of TGFβ, which further suppress STING expression and accelerate CRC progression. Notably, an inhibitor, 1-Hydroxybaccatin I, that disrupts the MKKS3-TRIM29 interaction, was identified, which, when combined with oxaliplatin, enhanced antitumour efficacy in vivo. Moreover, intratumoural administration of AAV8-shMKKS3 alongside oxaliplatin resulted in improved therapeutic outcomes.

## Materials and methods

### Cell culture and drugs

Human CRC cell lines (HT29, SW480, SW620, HCT116, RKO, DLD1) were obtained from ATCC (Rockville, MD, USA), whereas the normal colonic epithelial cell line NCM460 was sourced from INCELL Corporation (San Antonio, TX, USA). Fibroblasts were purchased from FUHENG Biology (Shanghai, China). Cells were cultured in RPMI 1640 or DMEM (Thermo Fisher Scientific, San Jose, CA, USA), supplemented with 10% fetal bovine serum (FBS) (ExCell Bio, Shanghai, China) in a 5% CO_2_ incubator at 37 °C. All cell lines used were cultured within 35 generations and tested negative for Mycoplasma throughout the study. The short tandem repeat profiling was used to verify the identity of cell lines. The TargetMol Natural Compound Library (30 mM), KU-55933, berzosertib and oxaliplatin were acquired from TargetMol (Boston, MA, USA) and dissolved in dimethyl sulfoxide (DMSO).

### Identification of long non-coding RNA-encoded proteins

Raw pan-cancer data were obtained from Clinical Proteomic Tumor Analysis Consortium (CPTAC) studies with the following IDs: PDC000120 (breast), PDC000411 (kidney), PDC000116 (colon), PDC000221 (head and neck), PDC000198 (liver), PDC000234 (lung), PDC000110 (ovary), PDC000341 (pancreas) and PDC000125 (uterus). The data were analysed using a reference library from a previous study. TMT datasets (breast, colon, head and neck, liver, lung, ovarian and uterine cancers) were processed with Proteome Discoverer (Thermo Scientific), whereas DIA datasets (kidney and pancreatic cancers) were analysed using Spectronaut (Biognosys, Switzerland). All samples were analysed in the study; there was no attrition. Proteins with no missing values in more than 30% of tumour samples or more than 30% of non-tumour samples were included and classified as lncRNA-encoded proteins.

### Western blotting analysis

Western blotting was conducted as previously described^21,22^. The primary antibodies used included GFP (Proteintech, Rosemont, IL, USA; 50430-2-AP; RRID:AB_11042881), Flag (Proteintech; 20543-1-AP; RRID:AB_11232216), TRIM29 (Proteintech; 17542-1-AP; RRID:AB_2272412), FAP (Cell Signaling Technology, Beverly, MA, USA; #52818; RRID:AB_3674735), α-SMA (Proteintech; 14395-1-AP; RRID:AB_2223009), λ-H2AX (Active Motif, Carlsbad, CA, USA; 39689; RRID:AB_2728764), HA (Cell Signaling Technology; #3724; RRID:AB_1549585), MSH2 (Proteintech; 15520-1-AP; RRID:AB_2144799), BRCA1 (Proteintech; 22362-1-AP; RRID:AB_2879090), ATM (Proteintech; 27156-1-AP; RRID:AB_2880780), H3K36me3 (Active Motif; 61021; RRID: AB_2614986), STING (Proteintech; 19851-1-AP; RRID:AB_10665370), Ki-67 (Proteintech; 27309-1-AP; RRID:AB_2756525), Ub (Proteintech; 10201-2-AP; RRID:AB_671515), TGFβ (Proteintech; 81746-2-RR; RRID:AB_3670503), Actin (Proteintech; 66009-1-Ig; RRID:AB_2687938), CD8 (Biolegend, San Diego, CA, USA; 100752; RRID:AB_2563057), CD4 (Biolegend; 100510; RRID:AB_312713), Granzyme B (Biolegend; 372208; RRID:AB_2687032) and CD69 (Biolegend; 164203; RRID:AB_2936560). Antibodies against MKKS3 were generated by immunizing rabbits with recombinant proteins (GL Biochem, Shanghai, China) and were purified via affinity chromatography using the corresponding recombinant proteins. Signals were visualized using Clarity Western ECL substrate (Bio-Rad, Hercules, CA, USA) and detected through autoradiographic film exposure.

### Plasmids, transfection and infection

The 5’UTR-ORF sequences of NR_072977 were cloned into a mutant EGFP vector with the start codon ATG mutated to ATT. The mutant constructs (5’UTR-ORFmut-GFPmut, 5’UTR-ORFmut-Flag) were generated using the Mut Express II Fast Mutagenesis Kit v2 (Vazyme, Nanjing, China). Small interfering RNAs (siRNAs) targeting TGF-β, TRIM29 and DENR, as well as doxycycline (DOX)-inducible ORF-knockdown plasmids, 5’UTR-ORF-Flag plasmids, stable ORF and ORFmut-overexpression plasmids, and TRIM29-expressing plasmids, were purchased from TransheepBio (Shanghai, China). The plasmids pcDNA3.1 (RRID:Addgene_128034)-TRIM29-mut1-HA, pcDNA3.1-TRIM29-mut2-HA, pcDNA3.1-TRIM29-mut3-HA and pcDNA3.1-TRIM29-mut4-HA were obtained via PCR amplification. Protocols for establishing stable cell lines and transfection were described previously^21^. The siRNAs were transfected into CRC cells using Lipofectamine 3000 (Thermo Fisher Scientific). Wild type and mutant MKKS3 5’UTR regions were cloned into the pGL3 vector (Promega, Madison, WI, USA; RRID:Addgene_48743). Dual luciferase reporter assays were conducted with the Dual-Luciferase Reporter Assay System (Promega). Cells were co-transfected with a luciferase plasmid containing the 5’UTR region of MKKS3 and a pRL-TK Renilla luciferase plasmid (RRID:Addgene_27163), and luciferase activity was measured according to the manufacturer’s instructions. Primer sequences and siRNA target sequences are listed in Supplementary Table 10.

### CRISPR/Cas9 screening

A CRISPR knockout pooled library targeting 314 lncRNAs was designed and constructed by Fubio Biological Technology Co., Ltd. (Shanghai, China). A library containing 10 single guide RNAs (sgRNAs) per lncRNA in addition to non-targeting sgRNAs was transduced into CRC cells. To conduct a CRISPR-Cas9 knockout screen, cells were transduced with the designed sgRNA library at a multiplicity of infection of approximately 0.3 to achieve coverage of at least 500-fold per guide RNA (gRNA). After transduction, cells were selected with puromycin (5 μg/ml) for 48 h and then subcutaneously injected into nude mice. To ensure adequate sgRNA representation, at least 6 × 10^6^ cells were maintained at any given time throughout the experiment. The experimental group received oxaliplatin (20 mg/kg) every 2 days. After 1 month, oxaliplatin-treated and control tumours were harvested, and genomic DNA was extracted using a DNA extraction and purification kit (Qiagen, Hilden, Germany) as previously described^14^. sgRNA inserts were amplified by PCR using NEBNext High Fidelity PCR Master Mix (NEB, Ipswich, MA, USA). For each sample, 10 μg of genomic DNA was used as input for PCR amplification. PCR products were then used to construct paired-end libraries with the Paired-End DNA Sample Prep Kit (Illumina Inc., San Diego, CA, USA). Deep sequencing was performed on a NovaSeq 6000 platform (Illumina Inc.) at Genedenovo Biotechnology Co., Ltd. (Guangzhou, China). Raw reads were filtered to obtain high-quality clean reads using the following criteria: 1) removing reads with ≥ 10% unidentified nucleotides (N); 2) removing reads with > 40% bases having Phred quality scores ≤ 20; 3) removing reads aligned to barcode adapters. Reads were mapped to the sgRNA library using Blastn (version 2.6.0 + ; parameter: -word_size 18). Resistance lncRNAs were identified from the sgRNA screen sequencing results using MAGeCK MLE analysis. MAGeCK MLE calculates a “beta score” for each targeted gene to measure the degree of selection upon gene perturbation, similar to the “log fold-change” measurement in differential expression analysis. A positive beta score indicates that the gene is a positively screened lncRNA, whereas a negative value indicates a negatively screened lncRNA. *P* value < 0.05 and negative beta scores were considered as significantly depleted sgRNAs.

### Cell viability assay

The WST-1 Cell Proliferation and Cytotoxicity Assay Kit (Beyotime Biotechnology, Shanghai, China) was used to assess CRC cell viability^23^. Briefly, 1 × 10^3^ cells were plated in 96-well plates, followed by treatment with oxaliplatin (0 μM, 10 μM, 20 μM) or 1-Hydroxybaccatin I (0 μM, 10 μM, 20 μM). WST-1 reagent was added, cells were incubated at 37 °C for 2 h and absorbance at 450 nm was measured using an automated microplate spectrophotometer (BioTek Instruments, Winooski, VT, USA).

### Colony formation assay

CRC cells transfected with MKKS3, MKKS3mut or TRIM29 siRNA were plated in 6-well or 12-well plates and treated with the indicated concentrations of oxaliplatin (0 μM, 10 μM, 20 μM) or 1-Hydroxybaccatin I (0 μM, 10 μM, 20 μM) for 2 weeks. Cells were then fixed with methanol, stained with 1% crystal violet and the colonies were quantified.

### Immunofluorescence staining

5’UTR-ORF or 5’UTR-ORFmut-fused GFP vectors were transfected into CRC cells, and GFP fluorescence was visualized by fluorescence microscopy (Nikon, Tokyo, Japan). Cells expressing 5’UTR-ORF-Flag or 5’UTR-ORFmut-Flag, treated with DOX (1 mg/ml) and the indicated concentrations of oxaliplatin, were fixed with 4% paraformaldehyde. After fixation, cells were permeabilized with 0.1% Triton X-100 for 15 min, followed by incubation with anti-MKKS3, anti-Flag (RRID:AB_11232216) or anti-λ-H2AX (RRID:AB_2728764) antibodies, after washing and blocking with bovine serum albumin. Secondary antibodies were used for staining in the dark, followed by DAPI staining (Beyotime Biotechnology) for immunofluorescence analysis.

### Generation of anti-MKKS3 antibody

The anti-MKKS3 antibody was generated by GL Biochem (Shanghai, China) through rabbit inoculation with a Keyhole limpet haemocyanin (KLH)-coupled specific peptide. The antibody was further purified by affinity chromatography using KLH-coupled peptide columns.

### Tissue microarray and multiplex immunohistochemical staining

A tissue microarray (TMA, Shanghai Outdo Biotech, China, HCol-Ade180Sur-04) consisting of 100 chemotherapy-treated CRC tissues (72 non-recurrent and 28 relapsed) and 72 normal tissues was utilized to evaluate the expression of MKKS3 and α-SMA. The immunohistochemistry (IHC) protocol was described previously^21^. The PANO 6-plex Immunohistochemistry Kit (Panovue, Beijing, China) facilitated the assessment of MKKS3 and α-SMA expression and their association with clinicopathological parameters. In summary, tissue slides underwent deparaffinization in xylene, rehydration in ethanol and blocking with blocking buffer (Panovue). Subsequently, they were incubated with primary antibodies for 60 min, washed with TBST, and treated with horseradish peroxidase-conjugated secondary antibodies for 10 min. Following a second incubation with primary antibodies, signals were visualized using Opal 520 TSA (1:200) and counterstained with DAPI for 10 min. Imaging was performed using a Vectra Polaris platform (PerkinElmer, Waltham, MA, USA), and analysis was conducted using HALO software (Indica Labs, Corrales, NM, USA).

### Immunohistochemistry

Tumour sample slides were blocked with normal serum and incubated overnight at 4 °C with anti-Ki-67 (RRID:AB_2756525), anti-STING (RRID:AB_10665370), anti-α-SMA (RRID:AB_2223009) or anti-MKKS3 antibodies. Biotinylated secondary antibodies and a peroxidase-conjugated avidin–biotin complex (Dako) were then applied. Visualization of staining was achieved using 3,3′-diaminobenzidine (Dako), followed by counterstaining with haematoxylin. Imaging was performed using the Vectra Polaris platform (PerkinElmer), with analysis conducted using HALO software.

### Mass spectrometry and bioinformatics analyses

Protein digestion and mass spectrometry were performed as previously described^24^. In brief, HT29 cells expressing MKKS3 and control cells were lysed, and proteins were digested with trypsin, vacuum-freeze-dried, resuspended in anhydrous acetonitrile and desalted using MonoTIP C18 Pipette Tips (GL Sciences, Tokyo, Japan). The resulting peptides were analysed using an Orbitrap Fusion Lumos mass spectrometer (Thermo Fisher Scientific). Raw data were processed with Proteome Discoverer (Thermo Fisher Scientific, RRID:SCR_014477) and Spectronaut (Omicsolution Co., Ltd., Shanghai, China) software, with a protein/peptide false discovery rate set at 1%.

### Gene set enrichment analysis

Differentially expressed proteins (DEPs) between the experimental and control groups were identified with a fold change ≥ 1.5 and *P* < 0.05. The DEPs were ranked by fold change values and subjected to gene set enrichment analysis (GSEA) to identify significantly enriched pathways. GSEA was performed using the GSEA function implemented in the R package “clusterProfiler” (v3.10.1). Enrichment plots were visualized using the gseaplot2 function from the “enrichplot package” (v1.2.0). The gene set c2.cp.kegg.v7.5.symbols.gmt was used as the reference gene set. Pathways with significant enrichment are shown based on the normalized enrichment score (NES) and *P* value. Datasets with |NES | > 1 and *P* < 0.05 were considered significantly enriched.

### Generation of conditioned medium from cell lines

CRC cell lines (HT29, SW480) and fibroblasts were cultured in RPMI1640 (Thermo Fisher Scientific) supplemented with 10% fetal bovine serum (FBS; ExCell Bio). When cells treated with DMSO, oxaliplatin, IFNβ, or conditioned medium (CM) reached 70–80% confluency, they were washed with PBS, and fresh RPMI1640/10% FBS was added. CM was collected 48 h later, centrifuged at 300 *g* for 5 min, filtered through 0.45-μm filters, and used fresh. Target cells were then treated with equal volumes of CM at a final concentration of 50% in complete medium (ratio 1:1) for 48 h, followed by subsequent assay analysis.

### Co-immunoprecipitation

Co-immunoprecipitation (Co-IP) was performed following established protocols^25^. Cell lysates were incubated with IgG (Santa Cruz Biotechnology) and protein A/G Sepharose beads (Invitrogen, Carlsbad, CA, USA) at 4 °C for 1 h. The supernatant was then mixed with a primary antibody and incubated overnight at 4 °C, followed by incubation with protein A/G Sepharose beads for an additional 4 h. After washing with PBS and lysis buffer, the beads were resuspended in 5 × SDS/PAGE loading buffer for subsequent western blotting analysis.

### Single-cell gel electrophoresis

Comet assays were conducted according to the manufacturer’s protocol (Trevigen, MD, USA). Briefly, cells were mixed with molten agarose at a 1:10 ratio, spread onto pre-warmed glass slides, and subjected to lysis in lysis solution (Trevigen) for 1 h. The slides were then immersed in 1 × neutral electrophoresis buffer for 30 min before electrophoresis at 4 °C for 45 min. After fixation, the cells were stained with SYBR Gold (Invitrogen), and the tail length and DNA intensity within the tail were quantified.

### Enzyme-linked immunosorbent assay

Enzyme-linked immunosorbent assay (ELISA) was employed to quantify the levels of IFNβ in the CM using the IFNβ ELISA kit (Abcam, Cambridge, MA, USA), following the manufacturer’s guidelines.

### Molecular docking

For molecular docking, the TRIM29 protein model was generated utilizing the Protein Data Bank (PDB; http://www.rcsb.org). The optimal binding pose of 1-Hydroxybaccatin I to TRIM29, determined by binding score, was selected for interaction analysis using Dock version 6.9 (https://dock.compbio.ucsf.edu/).

### In vivo experimental metastasis model

All animal experiments were approved by the Ethics Committee for Animal Experiments at Jinan University (20240703-01). Tumour xenograft experiments were performed according to established protocols. Six- to eight-week-old female BALB/c nude mice (RRID:IMSR_GPT:D000521) and C57BL/6 mice (RRID:IMSR_JAX:000664) were housed under standard conditions in compliance with institutional guidelines. A cell suspension (5 × 10^6^ for HT29, RRID:CVCL_0320; 2 × 10^6^ for MC38, RRID:CVCL_B288) was subcutaneously injected into the flanks of the mice. Tumour volume was calculated using the formula: V = (length × width^2^) / 2. Once tumours reached a diameter of approximately 5 mm, mice were randomly assigned to control and treatment groups. As shown in Fig. 2o, stably transfected MKKS3 cells were subcutaneously injected into nude mice, with xenograft measurements taken every 3 days to calculate tumour volumes. As shown in Fig. 7g, C57BL/6 mice were administered vehicle (0.5% CMC-Na), or 1-Hydroxybaccatin I (5 mg/kg), or oxaliplatin (5 mg/kg), or SB24011 (an inhibitor of the STING-TRIM29 interaction, 5 mg/kg), or a combination of 1-Hydroxybaccatin I and oxaliplatin, or a combination of SB24011 and oxaliplatin, once every 3 days. As shown in Fig. 8a: MC38 cells were subcutaneously injected into C57BL/6 mice, and the mice were intratumourally injected with AAV8-shMKKS3 (10^10^ or 10^11^) or control virus. Subsequently, mice received oral administration of either vehicle (0.5% CMC-Na) or oxaliplatin (5 mg/kg). To deplete CD8^+^ T cells, CD8a-depleting antibody (BioXcell, BE0061, RRID:AB_1125541, 200 μg) was injected twice a week, starting 2 weeks prior to treatment with drugs. Additionally, IFNAR1 (RRID:AB_2687723) neutralizing antibody was administered via intraperitoneal injection at a dose of 100 μg per mouse every 3 days. At the conclusion of the experiment, blood was collected for biochemical and haematological analysis, tumours were harvested for IHC, and liver, lung and kidney tissues were collected for histopathological analysis.

### Single-cell RNA sequencing

HT29 and MC38 xenograft tissues were transported in sterile culture dishes containing 10 ml 1× Dulbecco’s phosphate-buffered saline (Thermo Fisher, Cat. no. 14190144) on ice to remove residual tissue storage solution, followed by mincing on ice. Tissues were digested using dissociation enzyme solution consisting of 0.25% (w/v) Trypsin (Thermo Fisher, Cat. no. 25200-072) and 10 μg/ml DNase I (Sigma, Cat. no. 11284932001) dissolved in PBS containing 5% FBS (Thermo Fisher, Cat. no. SV30087.02). HT29 and MC38 xenograft tissues were dissociated at 37 °C with shaking at 50 rpm for approximately 40 min. Dissociated cells were repeatedly collected at 20-min intervals to maximize cell yield and viability. Cell suspensions were filtered through a 40-μm nylon cell strainer, and red blood cells were removed using 1× Red Blood Cell Lysis Solution (Thermo Fisher, Cat. no. 00-4333-57). Dissociated cells were washed with 1× Dulbecco’s phosphate-buffered saline containing 2% FBS. Cell viability was assessed using acridine orange/propidium iodide (AO/PI) fluorescent dye on a Countstar Fluorescence Cell Analyzer (Aber Instruments) prior to loading onto the 10× Chromium platform.

For 10x library preparation and sequencing, single-cell RNA sequencing (scRNA-seq) libraries were generated using the 10× Chromium Single Cell 3′ v3.1 kit following the manufacturer’s standard protocol. Briefly, cells were loaded onto the Chromium platform to generate Gel Beads-in-emulsion, where beads with unique molecular identifiers and cell barcodes were hybridized to polyadenylated RNA. After cell lysis, beads were retrieved for reverse transcription, followed by second-strand complementary DNA synthesis, adaptor ligation and library construction. Sequencing libraries were generated and quantified using a High Sensitivity DNA Chip (Agilent) on a Bioanalyzer 2100 and the Qubit High Sensitivity DNA Assay (Thermo Fisher Scientific). Libraries were sequenced on an Illumina Xplus platform using 2×150 chemistry. Alternatively, sequencing was performed on a DNBSEQ-T7 platform (PE150) using the DNBSEQ-T7RS Reagent Kit (FCL PE150) version 3.0. Sequencing and bioinformatic analysis were conducted by Majorbio Co., Ltd. (Shanghai, China).

For scRNA-seq data processing, raw reads were processed using Cell Ranger V7.1.0 with default parameters and aligned to the reference genome using STAR. FASTQs generated from Illumina sequencing output were aligned to the Mus_musculus (GRCm39) or Homo_sapiens (GRCh38) genome using the STAR algorithm. Gene-barcode matrices were generated by counting unique molecular identifiers and filtering non-cell barcodes. Downstream analysis was performed in Seurat (v4.1.1). Low-quality cells were excluded based on three criteria: number of detected transcripts, unique molecular identifiers, detected genes and mitochondrial gene percentage (mean ± 2-fold standard deviation). The normalized data (NormalizeData function in the Seurat package) was performed for extracting a subset of variable genes. Next, we integrated data from different samples after identifying ‘anchors’ between datasets using FindIntegrationAnchors and IntegrateData in the Seurat package, and visualization was performed using uniform manifold approximation and projection.

For clustering analysis, scaling, dimensionality reduction and clustering were applied to the specific dataset (restricted to one cell type). Differential expression analysis between clusters was performed using the Wilcoxon rank-sum test. Cell types were identified using SCINA and known marker genes. Differential expression genes (DEGs) between two different samples or clusters were identified using the FindMarkers function in Seurat with a likelihood ratio test. Functional enrichment analysis of Kyoto Encyclopedia of Genes and Genomes (KEGG) pathways was performed to identify significantly enriched terms and metabolic pathways compared with the whole-transcriptome background, with *P* value < 0.05. KEGG functional enrichment analysis was carried out using Python SciPy software.

### Measurement of CD8^+^ and CD4^+^ T cell subsets in tumour tissues

Tumour tissues were processed into single-cell suspensions to evaluate the proportion of central memory T cells within the tumour-infiltrating CD8^+^ and CD4^+^ T cell populations. CD4 (1:400, RRID:AB_312713) and CD8 (1:600, RRID:AB_2563057) expression was assessed by gating on the CD45^+^ cell population. For GZMB (1:100, RRID:AB_2687032) and CD69 (1:400, RRID:AB_2936560) detection, single-cell suspensions were stained with CD8 antibodies (RRID:AB_2563057), followed by washing with PBS, fixation and permeabilization using the BD kit (00-5523-00, eBioscience, Vienna, Austria) for intracellular staining. The expression of GZMB and CD69 was then analysed within the CD8^+^ population.

### Quantitative real-time-PCR

Total RNA was extracted using TRIzol (Thermo Fisher Scientific), and complementary DNA was synthesized with the PrimeScript II First-Strand complementary DNA Synthesis Kit (Takara, Dalian, China). Quantitative real-time-PCR was performed as previously described^21^, utilizing SYBR Green qPCR SuperMix (TransGen, Beijing, China) on a CFX Opus Real-Time PCR System (Bio-Rad). The relative expression of RNA was quantified using the following primers: MKKS3 forward 5′-CCTTCGGAACTTGTGGAGAGAC-3′, reverse 5′-ATCTTGCTCAGGAATGTCGTCGT-3′; GAPDH forward 5′-TGACTTCAACAGCGACACCCA-3′, reverse 5′-CACCCTGTTGCTGTAGCCAAA-3′; ACTA2 forward 5′-AAAAGACAGCTACGTGGGTGA-3′, reverse 5′-GCCATGTTCTATCGGGTACTTC-3′; IL6 forward 5′-AGTGAGGAACAAGCCAGAGC-3′, reverse 5′-CATTTGTGGTTGGGTCAGG-3′; CXCL1 forward 5′-TTGCCTCAATCCTGCATCCC-3′, reverse 5′-GTTGGATTTGTCACTGTTCAGCAT-3′; CXCL2 forward 5′-CCAACCACCAGGCTACAGG-3′, reverse 5′-GCGTCACACTCAAGCTCTG-3′.

### Statistics and reproducibility

All in vitro experiments were conducted in triplicate. Statistical analyses were performed using GraphPad Prism, which facilitated two-tailed Student’s *t* tests, one-way/two-way analysis of variance and log-rank tests, with results presented as mean ± standard deviation (SD). Survival analysis was conducted using the Kaplan–Meier method alongside the log-rank test. Datasets for MKKS3 receiver operating characteristic (ROC) curves were obtained from the CPTAC (PDC000166), while Gene Expression Omnibus databases (GSE72970 and GSE69657 datasets are based on the platform of GPL570[HG-U133_Plus_2] Affymetrix Human Genome U133 Plus 2.0 Array) were utilized to evaluate MKKS3 expression in CRC. Error bars represent SD; statistical significance is denoted as *P < 0.05, **P < 0.01, ***P < 0.001 and ns indicates no significance.

## Results

### Cryptic translation of microproteins hidden in long non-coding RNAs in human cancers

**Fig. 1:**
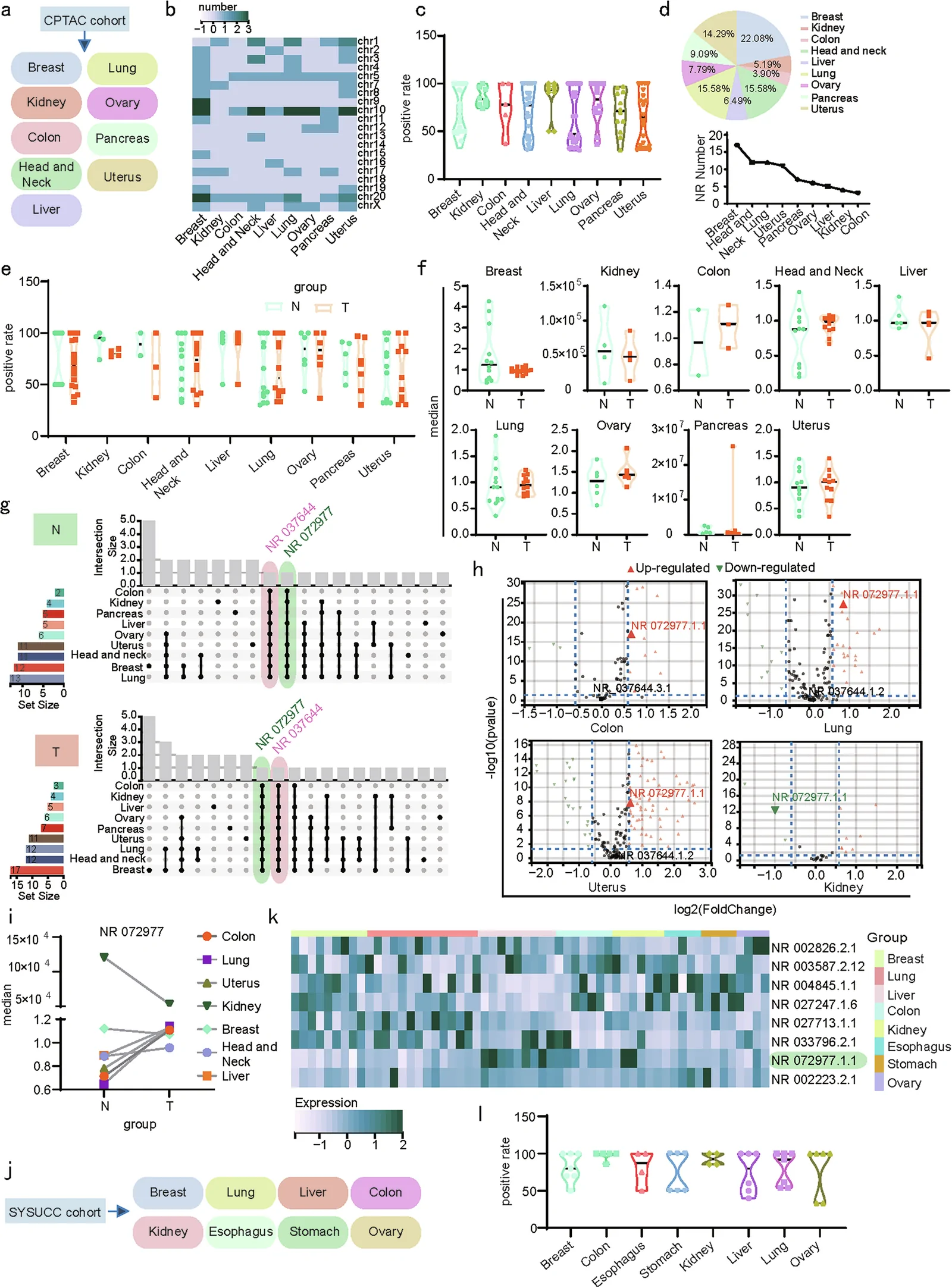
Expression profiles and distribution of potential proteins encoded by long non-coding RNAs across human cancers. **a** Schematic representation of the proteomic data source from the Clinical Proteomic Tumor Analysis Consortium (CPTAC), encompassing nine pan-cancer types. CPTAC study IDs were downloaded: PDC000120 (breast), PDC000411 (kidney), PDC000116 (colon), PDC000221 (head and neck), PDC000198 (liver), PDC000234 (lung), PDC000110 (ovary), PDC000341 (pancreas) and PDC000125 (uterus). **b** Chromosomal distribution of 314 potential proteins encoded by long non-coding RNAs, with proteins expressed in over 30% of tumour or non-tumour tissues within specific sample types considered identified. **c**, Positive rate distribution of potential proteins identified in the CPTAC cohort. **d–f** Comparisons of absolute protein counts (**d**) positive rates (**e**) and median expression levels (**f**) of potential proteins in tumour versus non-tumour tissues across nine cancer types. Proteins identified in more than 30% of tumour samples were considered expressed. **g** Universal expression patterns of potential proteins encoded by NR_037644 and NR_072977 in tumour (8/9 and 8/9 cancer types, respectively) and normal tissues (7/9 and 8/9 tissue types, respectively) from CPTAC data. **h** Volcano plot depicting differences in expression between cancer and non-cancer samples for identified potential proteins, with red indicating upregulation in cancer and blue indicating downregulation. **i** Expression levels of identified potential proteins across all nine cancer types, with colour coding for cancer type. **j** Clinical cohort data from Sun Yat-sen University Cancer Center (SYSUCC), including 58 post-surgical samples from patients with eight solid tumour types. **k** Heatmap of potential proteins identified in the SYSUCC cohort, with proteins expressed in over 30% of samples in each cancer type considered identified. **l** Positive rate distribution of identified potential proteins in the SYSUCC cohort. Bars represent standard deviation; *P < 0.05; **P < 0.01; ***P < 0.001; ns, not significant.

In our previous studies, ribosome-nascent chain complex sequencing (RNC-seq) was employed to identify 3,300 lncRNAs bound to ribosomes with active translation elongation across nine cancer cell lines. Among these, 314 lncRNAs were confirmed to encode unique microproteins through multiple reaction monitoring and parallel reaction monitoring^11^. In the present study, novel candidate proteins were screened using proteomic data from the CPTAC and Sun Yat-sen University Cancer Center databases, followed by functional characterization in CRC. Proteomic data from over 1,000 patients representing nine malignant tumour types in the CPTAC cohort were analysed (Fig. 1a; Supplementary Table 1). These 314 lncRNA-encoded proteins were distributed across various chromosomes (Fig. 1b; Supplementary Table 2). Among them, 21 potential proteins were detected in breast-cancer samples, with fewer detected in kidney-cancer (4/314) and colon-cancer (3/314) samples (Fig. 1c; Supplementary Table 3). Both the absolute numbers and positive rates of these proteins varied significantly across different cancer types (Fig. 1d–f; Supplementary Table 3). Notably, two potential proteins encoded by NR_037644 and NR_072977 were universally expressed in most tested cancer types (8/9 and 8/9, respectively), as well as in their normal counterparts (7/9 and 8/9, respectively) (Fig. 1g). Expression analysis across the nine cancers revealed that the NR_072977-encoded protein was upregulated in tumour tissues of colon, lung and uterine cancers, whereas it was downregulated in kidney cancer (Fig. 1h, i; Supplementary Table 4). Additionally, post-surgical samples from 58 patients with eight solid cancer types were collected from Sun Yat-sen University Cancer Center^26^ (Fig. 1j). Using the same strategy as in the CPTAC cohort, 8 potential proteins were identified, including the one encoded by NR_072977 (Fig. 1k, l; Supplementary Tables 5, 6). The absolute numbers and positive rates of these eight proteins varied considerably across different cancers (Fig. 1l; Supplementary Fig. 1), suggesting that lncRNA-encoded proteins may significantly influence cancer progression with different roles.

### Identification of key drivers of colorectal cancer chemoresistance

**Fig. 2:**
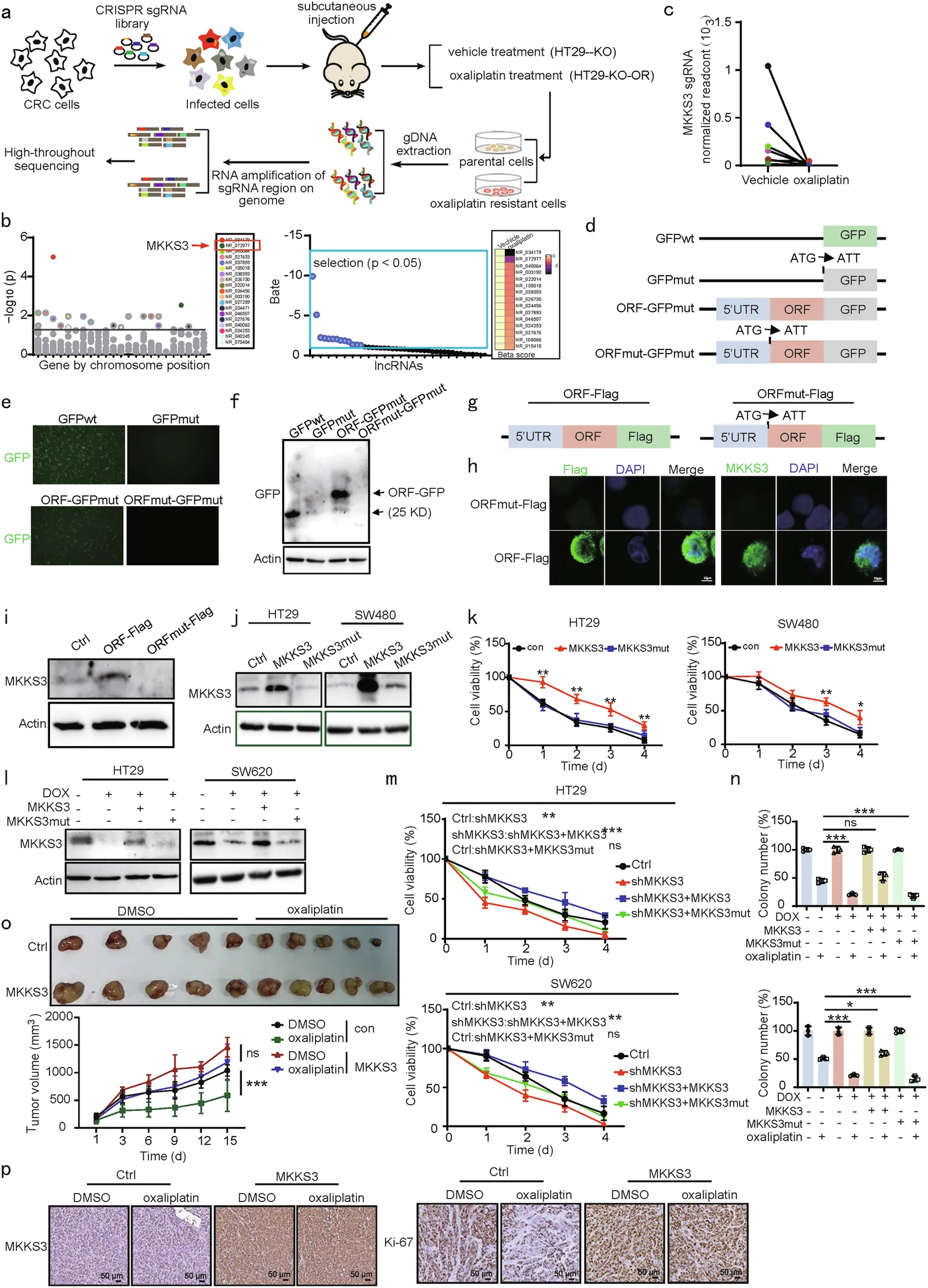
Identification of NR_072977 as a driver of colorectal cancer chemoresistance and validation of its encoded protein MKKS3. **a** Schematic overview of the CRISPR/Cas9 functional screening approach: HT29 cells transduced with a single guide RNA (sgRNA) library targeting 314 long non-coding RNAs were subcutaneously implanted into mice, followed by division into control or oxaliplatin treatment (20 mg/kg) groups. After 1 month, genome DNA (gDNA) was extracted from oxaliplatin-treated and parental cells for high-throughput sequencing. **b** Pilot screening results identifying NR_072977 as a key player in colorectal cancer chemoresistance. **c** Relative abundance of sgRNAs targeting NR_072977, showing a marked decrease in abundance in oxaliplatin-treated cells compared with control cells. **d** Schematic of plasmid constructs designed to verify open reading frame (ORF) translation: GFPmut (start codon ATG mutated to ATT) fused to 5’UTR-ORF or 5’UTR-ORFmut. **e** Fluorescence microscopy images showing MKKS3-GFP fusion protein expression in cells transfected with ORF-GFPmut or ORFmut-GFPmut constructs. **f** Western blotting analysis using anti-GFP antibodies confirms the predicted molecular weight of the MKKS3-GFP fusion protein in ORF-GFPmut-transfected cells (absent in ORFmut-GFPmut-transfected cells). **g** Schematic of Flag-tagged plasmid constructs: 5’UTR-ORF fused to Flag (ORF-Flag) or 5’UTR-ORFmut fused to Flag (ORFmut-Flag). **h** Immunofluorescence staining with anti-Flag antibodies detecting MKKS3-Flag fusion protein expression in ORF-Flag-transfected cells (not detected in ORFmut-Flag-transfected cells). Blue, DAPI; green, Flag. **i** Western blotting analysis showing detection of MKKS3-Flag expression by anti-MKKS3 antibody in ORF-Flag-transfected cells. **j**, **l** Successful establishment of stable MKKS3-overexpressing, MKKS3mut-overexpressing (**j**) and doxycycline (DOX)-inducible MKKS3-knockdown colorectal cancer cell lines (**l**). **k** HT29 and SW480 cells expressing MKKS3 or MKKS3mut were treated with oxaliplatin (20 μM) for 1–4 days, followed by WST-1 assay to assess cell proliferation. **m**, **n** Cells treated with DOX (1 mg/ml) for 48 h were re-expressed with MKKS3 or MKKS3mut and subsequently treated with oxaliplatin (20 μM) for 1–4 days. Oxaliplatin resistance was assessed via colony-formation (**m**) and WST-1 assays (**n**). **o** Control and MKKS3-overexpressing HT29 cells were subcutaneously injected into nude mice, followed by oxaliplatin (20 mg/kg) or vehicle treatment. Xenograft volumes were measured every 3 days. Tumour tissues were harvested post-treatment, and tumour volumes were compared. **p** MKKS3 and Ki-67 staining of tumour sections from nude mice. Scale bar, 50 μm. Bars, standard deviation; *P < 0.05; **P < 0.01; ***P < 0.001; ns, not significant.

To search for the microproteins potentially involved in CRC chemoresistance, a CRISPR/Cas9 library targeting the 314 lncRNAs was developed for functional screening. This library was introduced into HT29 cells, which were subsequently implanted subcutaneously into mice. The mice were divided into a control group and a drug-treated group receiving oxaliplatin with a dosage of 20 mg/kg. After 1 month, genomic DNA was extracted from the tumours for PCR amplification and high-throughput sequencing (Fig. 2a). Preliminary analysis identified NR_072977 as a candidate with functional relevance to CRC chemoresistance (Fig. 2b), where sgRNAs targeting NR_072977 showed a marked reduction in abundance in oxaliplatin-treated cells (Fig. 2c). Bioinformatics analysis revealed a 189-nucleotide open reading frame (ORF) within NR_072977, predicted to encode a protein consisting of 63 amino acids, named MKKS3 (Supplementary Fig. 2a). To validate the predicted ORF, plasmids were constructed to fuse GFPmut (with the start codon ATG mutated to ATT) to either the 5’ UTR-ORF (ORF-GFPmut) or the 5’ UTR-ORFmut (ORFmut-GFPmut) (Fig. 2d). The MKKS3-GFP fusion protein was successfully detected in cells transfected with ORF-GFPmut, but not in those transfected with ORFmut-GFPmut (Fig. 2e), with Western blotting confirming the expected molecular weight (Fig. 2f). Similarly, Flag-tagged constructs (ORF-Flag and ORFmut-Flag; Fig. 2g) showed MKKS3-Flag expression exclusively in ORF-Flag-transfected cells, but not in ORFmut-Flag-transfected cells (Fig. 2h, i). To further confirm MKKS3 translation from NR_072977, an anti-MKKS3 antibody was generated (Fig. 2i). Consistently, MKKS3-Flag fusion protein expression was not detected by the anti-MKKS3 antibody when the start codon ATG of the predicted ORF in NR_072977 was mutated (Fig. 2i). Mass spectrometry further confirmed the presence of the MKKS3 protein (Supplementary Fig. 2b). Collectively, these results indicate that NR_072977 can encode the MKKS3 protein.

To assess the role of MKKS3 in CRC chemoresistance, cell lines overexpressing MKKS3, MKKS3mut or DOX-inducible MKKS3 knockdown were established (Fig. 2j, l). WST-1 and colony-formation assays demonstrated that MKKS3 overexpression, but not MKKS3mut, significantly counteracted the anticancer effects of oxaliplatin (Fig. 2k; Supplementary Fig. 3a) in CRC cells. Conversely, MKKS3 knockdown resulted in reduced oxaliplatin resistance, and the effect can be restored by MKKS3 re-expression, but not by MKKS3mut (Fig. 2m, n; Supplementary Fig. 3b). In vivo, control and MKKS3-overexpressing cells were subcutaneously injected into nude mice, which were then orally administered oxaliplatin or vehicle control. As shown in Fig. 2o, tumour tissues were harvested for tumour volume comparison, with oxaliplatin treatment (20 mg/kg) significantly inhibiting tumour volume in the control group bearing HT29-con cells. Ki-67 staining further confirmed that MKKS3 facilitated the suppression of oxaliplatin-induced proliferation (Fig. 2p). These results suggest that the MKKS3 protein encoded by NR_072977 plays a critical role in promoting oxaliplatin resistance in CRC.

### Clinical significance of MKKS3 expression in colorectal cancer

**Fig. 3:**
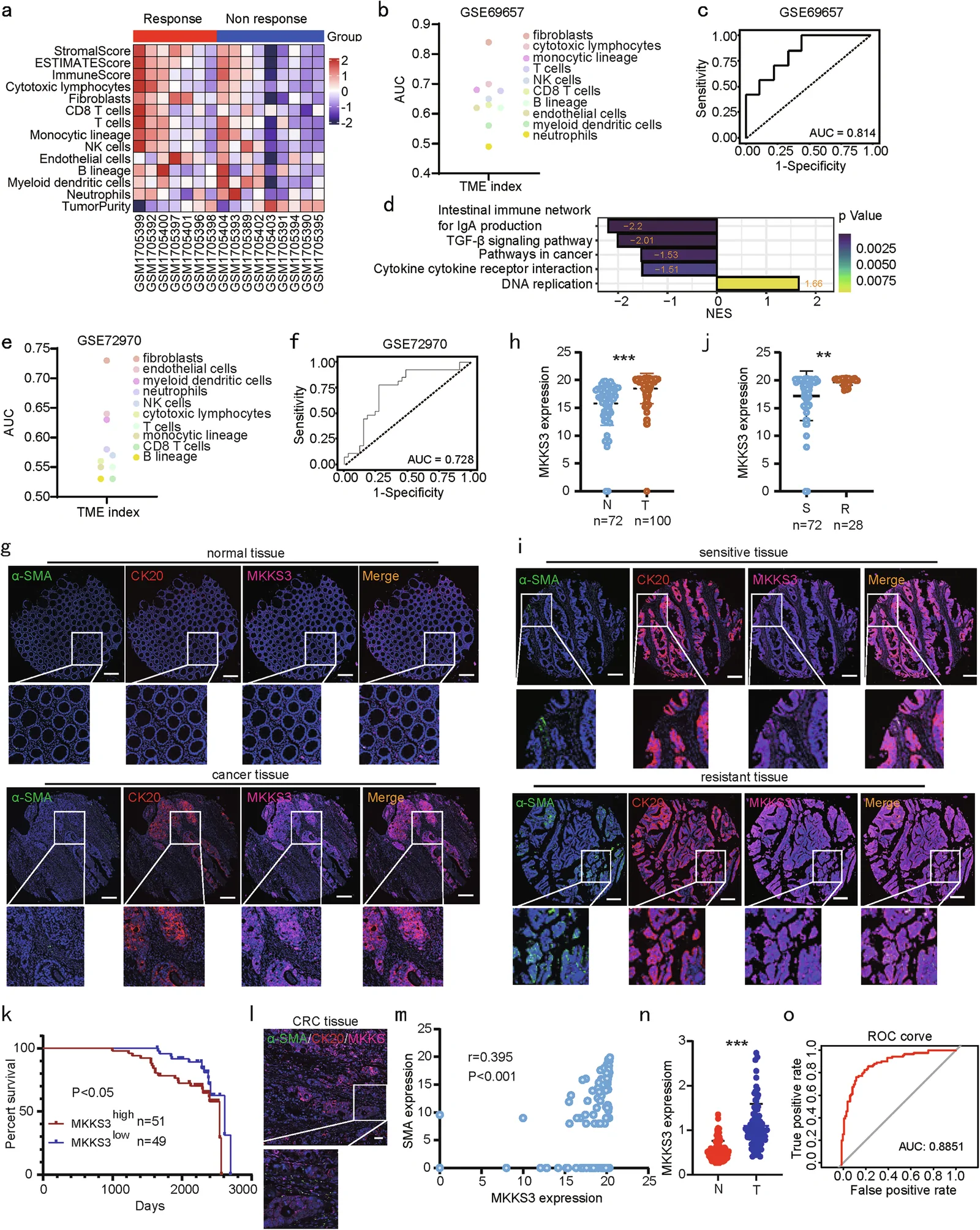
Clinical significance of MKKS3 expression in colorectal cancer. **a** Overview of the GEO dataset GSE69657, including 9 CRC samples in the “Non-response” group and 7 in the “Response” group to FOLFOX4 chemotherapy. **b**, **c** Correlation analysis of tumour microenvironment (TME) component abundance with chemotherapeutic response in GSE69657, identifying fibroblasts as the most significantly associated component (area under the curve (AUC) = 0.814). **d** Kyoto Encyclopedia of Genes and Genomes pathway analysis of differentially expressed genes (fold-change ≥ 1.5, *P* value < 0.05) from GSE69657 via CTR-DB (version 2.0, http://ctrdb.cloudna.cn/browse/dataset). **e**, **f** Correlation analysis of TME component abundance with chemotherapeutic response in GSE72970, highlighting fibroblasts as the most significantly associated component (AUC = 0.728). **g**, **h** Representative images and expression patterns of MKKS3 (violet), fibroblast marker α-SMA (green) and tumour cell marker CK20 (red) in 100 tumour and 72 normal tissue cases (**g**) showing higher MKKS3 expression in tumour tissues than in adjacent normal tissues (**h**). Scale bar, 200 μm. **i**, **j** Representative images and expression patterns of MKKS3, fibroblast marker α-SMA and tumour cell marker CK20 in 72 chemosensitive and 28 chemoresistant tissue cases (**i**) revealing higher MKKS3 expression in resistant tissues than in sensitive ones (**j**). Scale bar, 200 μm. **k** Kaplan–Meier survival analysis of 100 chemotherapy-treated patients with CRC stratified by MKKS3 expression (high vs low), indicating shorter survival in the MKKS3-high group. **l** Expression patterns of MKKS3 and α-SMA in CRC tissues. Scale bar, 50 μm. **m** Correlation analysis between MKKS3 and α-SMA expression in CRC tissues. **n** Comparison of MKKS3 protein expression levels between CRC and normal tissues using Clinical Proteomic Tumor Analysis Consortium (PDC000166) raw data, showing higher MKKS3 expression in CRC tissues. **o** Receiver operating characteristic (ROC) curve analysis of MKKS3 as a diagnostic marker for CRC, with an AUC of 0.885. Bars, standard deviation; *P < 0.05; **P < 0.01; ***P < 0.001; ns, not significant. NK, natural killer.

Key regulators and biological processes involved in CRC chemoresistance were investigated through analysis of the GEO dataset GSE69657, which included 9 CRC tissues in the “Non-response” group and 7 in the “Response” group following FOLFOX4 chemotherapy (Fig. 3a). TME component abundance was assessed from bulk transcriptomes. As shown in Fig. 3b, c, fibroblast abundance exhibited the strongest correlation with chemotherapeutic response (area under the curve = 0.814), consistent with the GSE72970 analysis emphasizing the role of fibroblasts in CRC chemotherapy resistance (Fig. 3e, f). KEGG analysis of DEGs (fold change ≥ 1.5, *P* value < 0.05; Supplementary Table 7) identified TME-associated pathways, including TGFβ signalling, cancer pathways, cytokine–cytokine receptor interaction and DNA replication, as crucial in CRC chemotherapy resistance (Fig. 3d). To examine the role of fibroblasts, multiplex immunohistochemistry was employed to detect MKKS3, the fibroblast marker α-SMA, and the tumour cell marker CK20 in a tissue microarray containing 100 CRC tumours with chemotherapy-related data and 72 normal tissue samples. Most tumour tissues showed elevated MKKS3 expression compared with adjacent normal tissues (Fig. 3g, h), with tumours receiving recurrent chemotherapy often exhibiting high MKKS3 levels (Fig. 3i, j). The 100 patients with CRC who underwent chemotherapy were divided into MKKS3-high and -low groups based on protein expression. Kaplan–Meier survival analysis revealed poorer survival in patients with high MKKS3 expression than in those with low MKKS3 expression (Fig. 3k), indicating a better chemotherapy response in the low MKKS3 expression group. Notably, MKKS3 expression correlated positively with α-SMA levels (Fig. 3l, m), suggesting a potential link between MKKS3 and fibroblast activity. Further analysis of the CPTAC data (PDC000166) confirmed higher MKKS3 expression in tumour samples than in normal tissues (Fig. 3n; Supplementary Fig. 4). Additionally, ROC curve analysis (area under the curve = 0.885) supported MKKS3 as a promising diagnostic marker for CRC (Fig. 3o). Together, these findings position MKKS3 expression as a prognostic biomarker for CRC chemotherapy outcomes.

### Downregulation of MKKS3 promotes DNA damage repair

**Fig. 4:**
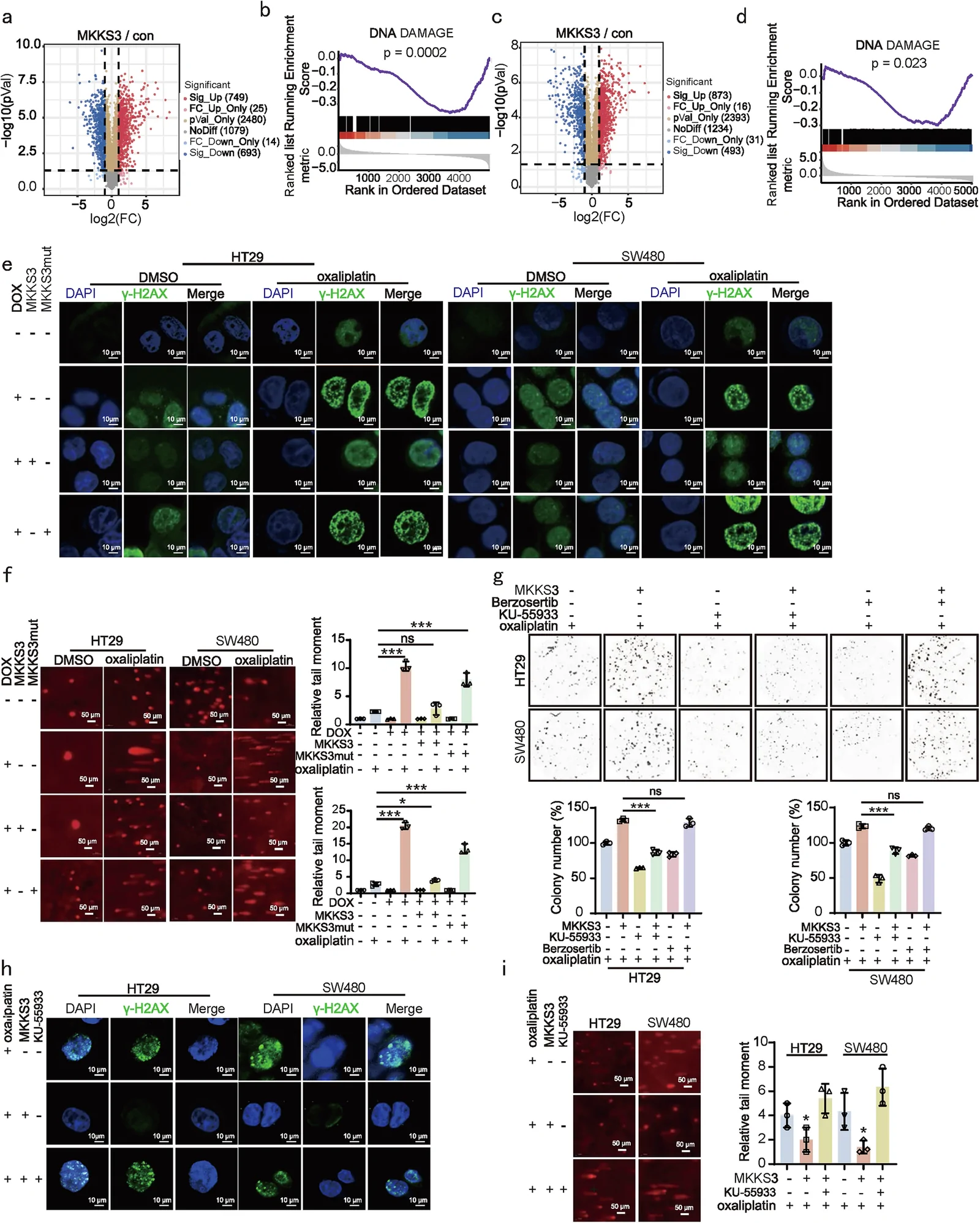
MKKS3 regulates DNA damage contributing to oxaliplatin resistance in colorectal cancer cells. **a**, **c** Volcano plots showing differentially expressed proteins in MKKS3-overexpressing HT29 (a) and SW480 (c) cells compared to control colorectal cancer cells, identified via DIA-based proteomics. In MKKS3-overexpressing HT29, a total of 1442 proteins (749 upregulated, 693 downregulated) and in MKKS3-overexpressing SW480, 1,366 proteins (873 upregulated, 493 downregulated) were significantly regulated. Fold change ≥ 1.5, *P* value < 0.05. **b**, **d** Kyoto Encyclopedia of Genes and Genomes pathway analysis of differentially expressed proteins regulated by MKKS3 in HT29 (**b**) and SW480 (**d**) cells, showing significant enrichment in the DNA damage signalling pathway in MKKS3-mediated oxaliplatin resistance. **e**, **f** Assessment of oxaliplatin-induced DNA damage in MKKS3-overexpressing, MKKS3mut-overexpressing and control cells via comet assay (**f**, scale bar, 10 μm) and γ-H2AX immunofluorescence staining (**e**, scale bar, 50 μm). Blue, DAPI; green, γ-H2AX. **g** Colony-formation assay evaluating the effects of ATM inhibitor (KU-55933) and ATR inhibitor (berzosertib) on MKKS3-mediated oxaliplatin resistance. Cells were pretreated with KU-55933 (5 μM, 6 h) or berzosertib (2 μM, 6 h), then treated with oxaliplatin (20 μM, 48 h). **h**, **i** Comet assay (**h**, scale bar, 50 μm) and γ-H2AX immunofluorescence staining (**i**, scale bar, 10 μm) demonstrating that KU-55933 negated the effect of MKKS3 on oxaliplatin-induced DNA damage. Cells were pretreated with KU-55933 (5 μM, 6 h), followed by oxaliplatin (20 μM, 48 h) treatment. Bars, standard deviation; *P < 0.05; **P < 0.01; ***P < 0.001; ns, not significant.

To explore the molecular mechanism underlying the role of MKKS3 in CRC chemoresistance, DIA-based proteomics was employed to identify DEPs in MKKS3-overexpressing HT29 and SW480 cells. The PLGEM algorithm revealed a strong linear correlation between the mean and SD of the quantitative protein values (correlation coefficients: 0.972, 0.944), with Q-Q plots confirming that residuals followed a normal distribution (Supplementary Fig. 5a–d). In triplicate experiments, 5040 proteins were identified in both cell lines (Fig. 4a, c), with 1,442 proteins (749 upregulated, 693 downregulated) and 1,366 proteins (873 upregulated, 493 downregulated) significantly modulated by MKKS3 (fold change ≥ 1.5, *P* value < 0.05; Supplementary Table 8) in HT29 and SW480 cells, respectively. Pathway analysis consistently identified the DNA damage signalling pathway as pivotal for MKKS3-mediated oxaliplatin resistance (Fig. 4b, d), a finding being further supported by Fig. 3d. To examine the role of MKKS3 in DNA damage response, a series of functional assays were conducted. The level of oxaliplatin-induced DNA damage was significantly lower in MKKS3-overexpressing cells, as assessed by comet and γ-H2AX IF staining and Western blotting (Fig. 4e, f; Supplementary Fig. 5e). MKKS3mut did not alter oxaliplatin-induced DNA damage (Fig. 4e, f). Considering the critical role of ATM and ATR in DNA damage repair^27,28^, the ATM inhibitor KU-55933 and the ATR inhibitor berzosertib were employed. The colony-formation assay revealed that KU-55933, but not berzosertib, reversed MKKS3-driven oxaliplatin resistance, suggesting that ATM activity is crucial for MKKS3-mediated DNA repair (Fig. 4g). Additionally, KU-55933 reversed the suppression of oxaliplatin-induced DNA damage caused by expression of MKKS3 (Fig. 4h, i), thereby confirming that ATM is essential for MKKS3-regulated oxaliplatin-induced DNA damage.

### MKKS3 protein interacts with TRIM29 to regulate DNA damage and STING expression

**Fig. 5:**
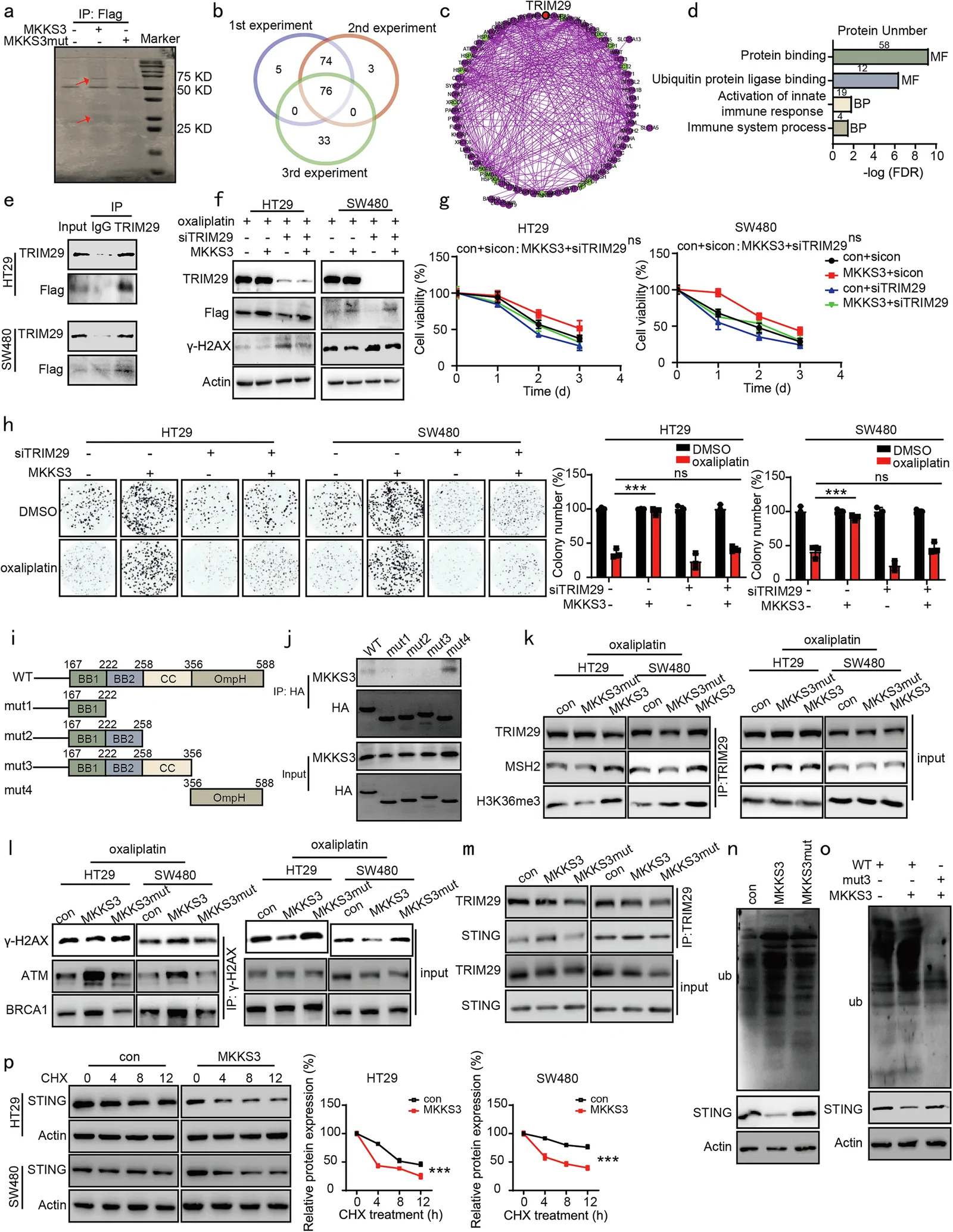
MKKS3 interacts with TRIM29 to regulate DNA damage repair and STING expression. **a** Coomassie blue staining revealing proteins that specifically bind to MKKS3; highlighted bands are marked with red arrows. **b** Venn diagram showing 76 proteins consistently identified in triplicate immunoprecipitation-mass spectrometry experiments as MKKS3-interacting candidates. **c**, **d** Functional annotation and pathway analysis of MKKS3-interacting proteins, demonstrating significant enrichment in processes such as protein binding, ubiquitin-protein ligase binding, activation of innate immune response and immune system functions. **e** Co-immunoprecipitation (Co-IP) assay confirming the interaction between MKKS3 and TRIM29 in colorectal cancer (CRC) cells. **f–h** MKKS3-overexpressing and control CRC cells were transfected with siRNA targeting TRIM29 (100 nM, 12 h), followed by oxaliplatin treatment (20 μM, 48 h). Western blotting, cell proliferation (**g**), and colony-formation (**h**) assays were performed to assess γ-H2AX expression and oxaliplatin resistance. **i** Mapping of TRIM29 truncation mutants with deletions in BB1, BB2, CC or OmpH domains. **j** TRIM29 and its truncation mutants (mut1, mut2, mut3) fused with HA plasmids were transfected into CRC cells. Co-IP results show reduced interaction between MKKS3 and TRIM29 mutants lacking the OmpH domain (mut1, mut2, mut3), indicating that the OmpH domain is crucial for binding. **k** Co-IP analysis showing that MKKS3 enhances TRIM29 binding to H3K36me3 and MSH2. In contrast, MKKS3mut (start codon ATG mutated to ATT) failed to enhance these interactions. **l** Co-IP assay demonstrating that MKKS3 promotes the interaction between ATM, γ-H2AX and BRCA1, whereas MKKS3mut (with start codon ATG mutated to ATT) did not exhibit this effect. **m** Co-IP analysis revealed that MKKS3, but not MKKS3mut (with start codon ATG mutated to ATT), enhanced the interaction between TRIM29 and STING. **n** Ubiquitination assay showing that MKKS3 (but not MKKS3mut) increases STING polyubiquitination. **o** Ubiquitination assay demonstrating that mut3, which lacks the OmpH domain, reduces STING polyubiquitination compared with wild type (WT) TRIM29. **p** Cycloheximide (CHX) chase assay showing that MKKS3 decreases the half-life of STING protein, indicating enhanced STING degradation. CHX, 50 μg/ml. Bars, standard deviation; *P < 0.05; **P < 0.01; ***P < 0.001; ns, not significant.

To investigate the underlying functional mechanisms, immunoprecipitation-mass spectrometry was employed to identify proteins interacting with MKKS3 (Fig. 5a). A total of 76 proteins were consistently detected across three independent replicates (Fig. 5b). Functional annotation of these interactors revealed that MKKS3 may regulate processes such as protein binding, ubiquitin ligase activity, innate immune activation and immune signalling through interactions with key regulatory molecules (Fig. 5c, d). Among these candidates, TRIM29 stood out owing to its known involvement in DNA damage repair^29^ and STING degradation-mediated suppression of innate immunity^30^. Co-IP assays further confirmed the interaction between MKKS3 and TRIM29 (Fig. 5e). To determine whether TRIM29 mediates MKKS3 function, CRC cells were transfected with TRIM29-targeting siRNA (siTRIM29), followed by assessments of cell proliferation and oxaliplatin resistance. Knockdown of TRIM29 abolished MKKS3-induced downregulation of γ-H2AX, decreased DNA damage and enhanced resistance to oxaliplatin (Fig. 5f–h; Supplementary Fig. 6a, b), suggesting that MKKS3 enhances DNA repair and oxaliplatin resistance through its interaction with TRIM29. TRIM29 contains several recognized substrate-binding domains, including BB1, BB2, CC and OmpH. To identify the essential domains for binding, TRIM29 was truncated with an HA tag and co-expressed with MKKS3. The results showed that deletion of the OmpH domain (mut1, mut2, mut3) abolished MKKS3-TRIM29 binding, whereas deletions of other domains had no discernible effect, highlighting OmpH as the critical interaction motif (Fig. 5i, j).

Given the role of TRIM29 as a scaffold in DNA repair complex assembly and its coordination with histone modifications, DNA repair protein complex and ATM on γ-H2AX nucleosomes, this study examined whether MKKS3 influences these interactions. Results showed that MKKS3 significantly enhanced TRIM29 binding to H3K36me3 and MSH2 (Fig. 5k). Additionally, MKKS3 enhanced the binding of ATM, γ-H2AX and BRCA1 (Fig. 5l). Furthermore, Co-IP assays revealed that in TRIM29-knockout HT29 and SW480 cells, MKKS3 could no longer regulate the assembly of the DNA repair complex, suggesting that MKKS3 promotes DNA damage repair through TRIM29 (Supplementary Fig. 6c). Considering the established interaction between TRIM29 and STING, the impact of MKKS3 on this association was also investigated. Co-IP assays revealed that MKKS3, but not MKKS3mut, enhanced the interaction between TRIM29 and STING (Fig. 5m). MKKS3, but not MKKS3mut, significantly increased the ubiquitination of STING (Fig. 5n), whereas the OmpH domain deficiency in TRIM29 did not affect the MKKS3-regulated STING ubiquitination (Fig. 5o). Moreover, cycloheximide experiments demonstrated that MKKS3 significantly decreased the half-life of STING protein, indicating that MKKS3 promotes STING degradation (Fig. 5p). Collectively, these results suggest that MKKS3 regulates DNA damage repair and STING ubiquitination through interaction with TRIM29.

### MKKS3-treated colorectal cancer cells upregulate the activity of fibroblasts and drive iCAF-to-myCAF conversion in a paracrine manner

**Fig. 6:**
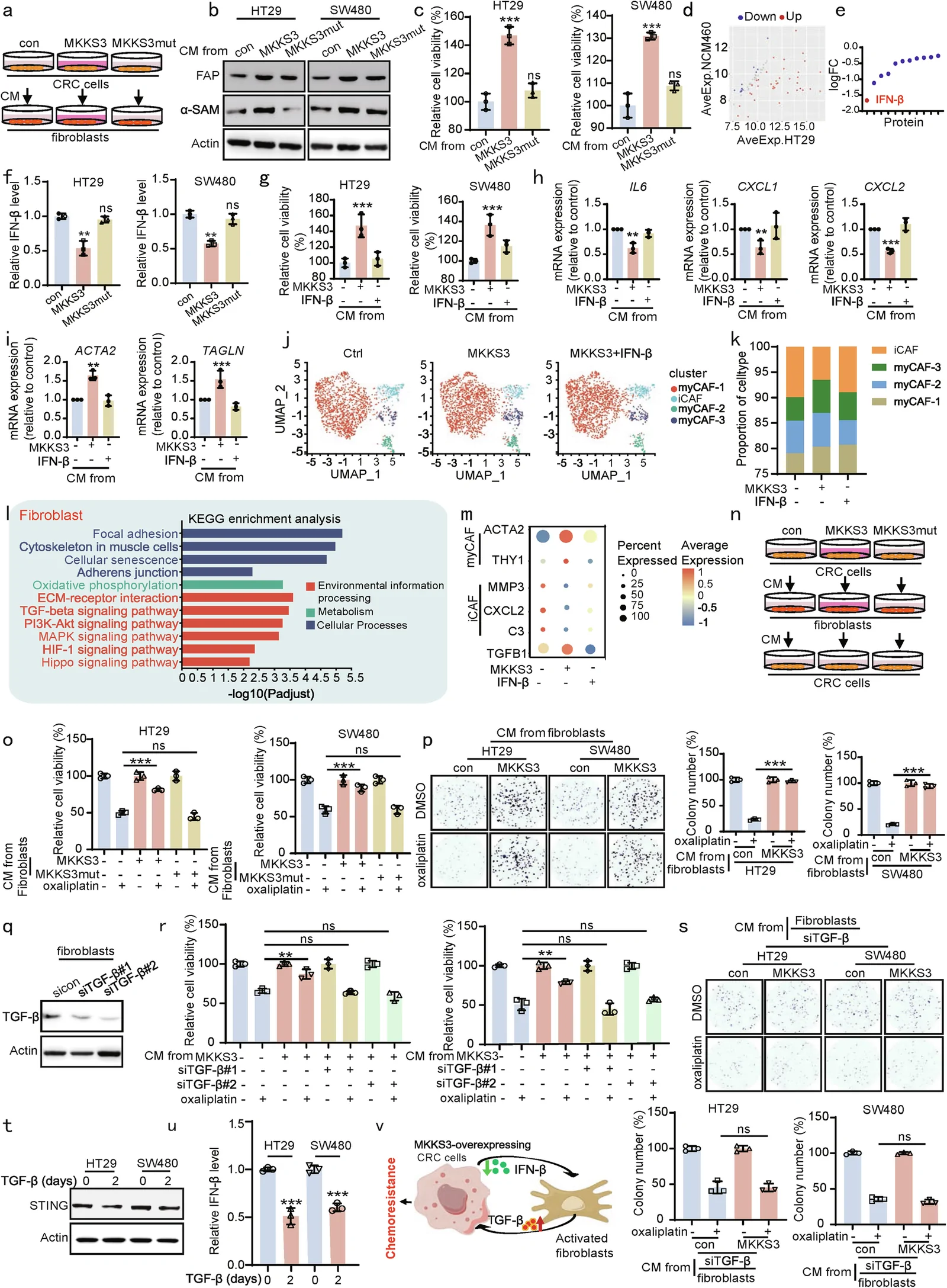
MKKS3-overexpressing colorectal cancer cells promote fibroblast activation via paracrine signalling and establish a feedback loop with fibroblasts. **a** Diagram and quantification of the collection of conditioned medium (CM) from the indicated colorectal cancer (CRC) cells to attract fibroblasts. **b** Western blotting analysis of cancer-associated fibroblasts (CAF) markers (α-SMA and FAP) in fibroblasts treated with CM (24 h) from MKKS3-overexpressing, MKKS3mut-overexpressing or control CRC cells. **c** WST-1 assay demonstrating enhanced fibroblast proliferation upon treatment with CM from MKKS3-overexpressing CRC cells. **d** Antibody microarray analysis of 80 cytokines in CM from CRC cells and the normal colonic epithelial cell line NCM460, identifying 49 MKKS3-regulated cytokines (38 upregulated, 11 downregulated). **e** Highlighted downregulated cytokine IFNβ (a STING target gene) from the antibody microarray results. **f** Enzyme-linked immunosorbent assay confirming IFNβ secretion in CM from MKKS3- and MKKS3mut-overexpressing CRC cells. **g–i** IFNβ protein (10 U/ml) was added to CM from MKKS3-overexpressing, MKKS3mut-overexpressing and control CRC cells to attract fibroblasts. WST-1 assay showing fibroblast cell viability (**g**) and quantitative PCR analysis of iCAF and myCAF-related markers (**h**, **i**). **j**, **k** Nude mice were subcutaneously co-injected with fibroblast cells together with either MKKS3-overexpressing HT29 cells or control cells. Subsequently, mice bearing MKKS3-overexpressing HT29 tumours were intraperitoneally administered with IFNβ protein (25 µg; every 3 days) or vehicle. Then, single-cell RNA sequencing was performed on fibroblasts isolated from subcutaneous xenograft HT29 tumours. The cell landscape was visualized using uniform manifold approximation and projection, analysing cells from HT29 tumours. All populations were annotated by cell type (**j**). Proportional distribution of cell types among fibroblasts is shown in **k**. **l** Single-cell RNA sequencing data of fibroblasts from HT29 tumours, and Kyoto Encyclopedia of Genes and Genomes analysis revealed significant pathway enrichment in fibroblasts. **m** Expression of myCAF markers (ACTA2, THY1), iCAF markers (MMP3, CACL2, C3) and TGFB1 of fibroblasts. **n** Schematic of the co-culture system: fibroblasts co-cultured with MKKS3-overexpressing, MKKS3mut-overexpressing or control CRC cells, followed by collection of fibroblast-derived CM for functional assays. **o**, **p** WST-1 (**o**) and colony-formation assays (**p**) demonstrating oxaliplatin resistance in CRC cells (20 μM, 48 h) treated with CM from fibroblasts co-cultured with MKKS3-overexpressing, MKKS3mut-overexpressing or control cells for 24 h. **q** TGFβ expression was knocked down in fibroblasts using siRNA targeting TGFβ (100 nM, 48 h). **r**, **s** WST-1 (**r**) and colony-formation assays (**s**) to evaluate the effect of TGFβ knockdown in fibroblasts on paracrine-mediated oxaliplatin resistance. **t** Western blotting showing that TGFβ treatment (5 ng/ml, 48 h) reduced STING expression in HT29 and SW480 cells. **u**, Enzyme-linked immunosorbent assay indicating decreased IFNβ secretion upon TGFβ treatment (5 ng/ml, 48 h) in CRC cells. **v** Schematic model illustrating the positive feedback loop between CRC cells and fibroblasts. Bars, standard deviation; *P < 0.05; **P < 0.01; ***P < 0.001; ns, not significant.

**Fig. 7:**
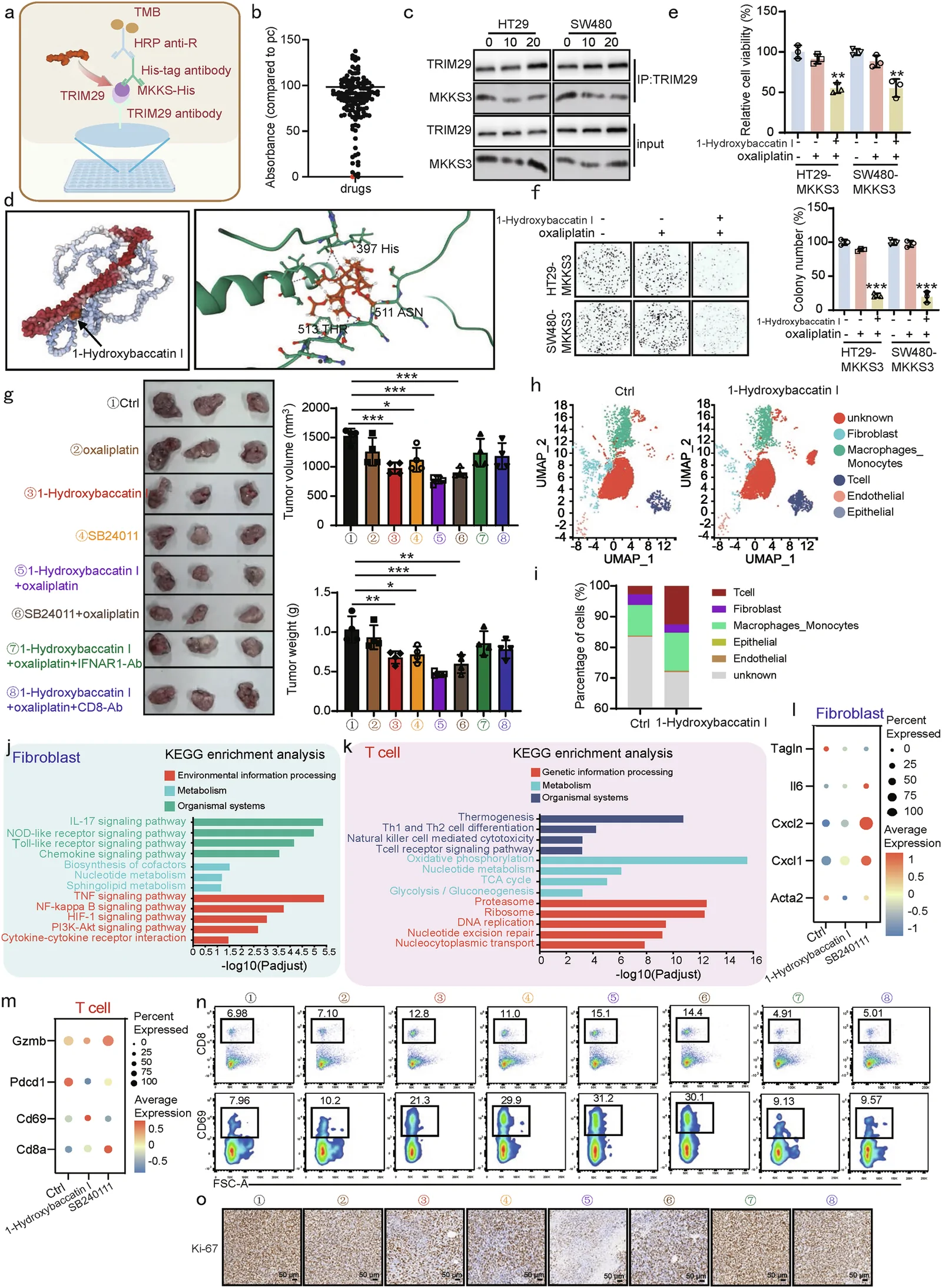
1-Hydroxybaccatin I enhances oxaliplatin sensitivity in colorectal cancer by disrupting the MKKS3–TRIM29 interaction and activating antitumour immunity. **a** Enzyme-linked immunosorbent assay-based drug-screening system utilizing a library of 150 plant natural products (10 μM). **b** Absorbance measurements in wells treated with different drugs. NC, negative control. **c** Co-immunoprecipitation assay in HT29 and SW480 cells treated with 1-Hydroxybaccatin I, showing a significant disruption of the MKKS3–TRIM29 interaction. **d** Molecular docking analysis predicting the binding of 1-Hydroxybaccatin I to TRIM29, highlighting key residues in the OmpH domain of TRIM29 (critical for the MKKS3–TRIM29 interaction) involved in the binding. **e**, **f** MKKS3-overexpressing colorectal cancer cells were treated with oxaliplatin (10 μM, 48 h) and 1-Hydroxybaccatin I (10 μM, 48 h), followed by WST-1 and colony-formation assays to evaluate colorectal cancer cell viability and colony-forming ability. **g** Representative images of excised MC38 tumours from control (Ctrl) and treatment groups. Treatment groups include oxaliplatin (5 mg/kg), 1-Hydroxybaccatin I (5 mg/kg), SB240111 (5 mg/kg), 1-Hydroxybaccatin I + oxaliplatin, SB240111 + oxaliplatin, 1-Hydroxybaccatin I + oxaliplatin + IFNAR1-Ab (100 μg per mouse), and 1-Hydroxybaccatin I + oxaliplatin + CD8α-Ab (200 μg per mouse). Tumour volume and weight were compared across the eight groups (left). **h**, **i** The cell landscape was visualized using uniform manifold approximation and projection, analysing cells from MC38 tumours. All populations were annotated by cell type (**h**). Proportional distribution of cell types among fibroblasts is shown in **i**. **j**, **k** Kyoto Encyclopedia of Genes and Genomes (KEGG) analysis revealed significant pathway enrichment in fibroblasts (**j**) and T cells (**k**). **l** Expression of myCAF markers (Tagln, Acta2) and iCAF markers (Il6, Cxcl1, Cxcl2) of fibroblasts. **m** Expression of Gzmb, Pdcd1, Cd69 and Cd8a of T cells. **n** Flow cytometry analysis of activation (CD69^+^) and infiltration of CD8^+^ T cells in tumour lysates. **o** Ki-67 staining of tumour sections. Scale bar, 50 μm. Bars, standard deviation; *P < 0.05; **P < 0.01; ***P < 0.001; ns, not significant.

Given the critical role of fibroblasts in cancer progression, MKKS3-overexpressing CRC cells might secrete cytokines that modulate fibroblasts via paracrine signalling. Western blotting analysis revealed that conditioned medium (CM) from MKKS3-overexpressing CRC cells, but not MKKS3mut-overexpressing cells, significantly increased the expression of CAF markers, including α-smooth muscle actin (α-SMA) and fibroblast activation protein (FAP) (Fig. 6a, b). This effect was accompanied by enhanced fibroblast proliferation (Fig. 6c), indicating effective fibroblast activation. Interestingly, we found that CM from MKKS3-overexpressing CRC cells induced the expression of several myofibroblasts (myCAF) markers, including ACTA2, TAGLN (Supplementary Fig. 7a), and reduced Inflammatory CAFs (iCAF) markers including IL6, CXCL1 and CXCL2 (Supplementary Fig. 7b), suggesting that MKKS3 drives iCAF-to-myCAF conversion in vitro. To investigate the underlying mechanisms, an 80-cytokine antibody microarray was used to compare CM from CRC cells and a normal colonic epithelial cell line (NCM460). The analysis identified 49 regulated cytokines, including 38 upregulations and 11 downregulations (Fig. 6d, e). Among the downregulated cytokines, IFNβ, a STING target gene with known immune-activating roles, was selected for further investigation (Fig. 6e; Supplementary Table 9). ELISA confirmed that MKKS3, but not MKKS3mut, negatively regulated IFNβ secretion in CRC cells (Fig. 6f). Importantly, the addition of IFN-β protein to cancer-cell-derived CM abolished the MKKS3-induced iCAF-to-myCAF conversion, and proliferation (Fig. 6f–i), highlighting the critical role of IFN-β in this paracrine signalling. To further elucidate the role of MKKS3 in the fibroblast activation in vivo, mice were subcutaneously co-injected with fibroblasts together with either MKKS-overexpressing HT29 cells or control cells. Subsequently, mice bearing MKKS3-overexpressing HT29 tumours were intraperitoneally administered with IFNβ protein or vehicle. Then, scRNA-seq was performed on fibroblasts isolated from subcutaneous xenograft HT29 tumours. Uniform manifold approximation and projection analysis distinguished four major clusters of CAFs (Fig. 6j; Supplementary Fig. 7c). Three of these clusters consistently showed high expression of myCAF markers, including COL8A1, COL11A1 and TAGLN, and were collectively annotated as myCAF subtypes (Supplementary Fig. 7c, d). Consistent with the results in vitro, MKKS3 drove iCAF-to-myCAF conversion, and this effect was abolished by treatment with IFNβ protein (Fig. 6k). KEGG analysis indicated significant enrichment of pathways related to environmental information processing, such as TGFβ signalling, in fibroblasts (Fig. 6l; Supplementary Table 11). Subsequently, the TGFB1 expression level was analysed, the results showed that MKKS3 markedly increased TGFB1 expression and this effect was abolished by IFNβ treatment (Fig. 6m; Supplementary Fig. 7e). Furthermore, myCAF markers (ACTA2 and THY1) were elevated, whereas iCAF markers (CXCL2, MMP3 and C3) were reduced in MKKS3-overexpressing tumours, and this effect was reversed by IFNβ (Fig. 7m; Supplementary Fig. 7e). Together, MKKS3 drove iCAF-to-myCAF conversion through IFNβ in vivo and in vitro. To assess the feedback from fibroblasts to cancer cells, a co-culture system was established using fibroblasts and MKKS3/MKKS3mut-overexpressing or control CRC cells (Fig. 6n). CM from fibroblasts co-cultured with MKKS3-overexpressing cells significantly enhanced CRC oxaliplatin resistance compared with CM from MKKS3mut-overexpressing or control co-cultures (Fig. 6o, p). Given that activated fibroblasts secrete elevated levels of TGFβ, TGFβ knockdown in fibroblasts attenuated their paracrine-mediated enhancement of oxaliplatin resistance in CRC cells (Fig. 6q–s). Furthermore, TGFβ inhibited STING expression and IFNβ secretion in CRC cells (Fig. 6t, u). Collectively, these data suggest that activated fibroblasts inhibit STING in CRC cells via secreted TGFβ, thereby reducing IFNβ secretion and enhancing fibroblast activity. This forms a feedback loop between CRC cells and fibroblasts. The role of IFNβ and TGFβ in this crosstalk is illustrated in Fig. 6v.

### 1-Hydroxybaccatin I enhances the sensitivity of colorectal cancer cells to oxaliplatin by disrupting MKKS3–TRIM29 interaction

Given the pivotal role of the MKKS3–TRIM29 interaction in CRC oxaliplatin resistance, an ELISA-based drug screening system was developed to identify inhibitors of this interaction. Plant natural products are a promising source of anticancer agents owing to their inherent anticancer properties, stability and clinical safety. Using a library of 150 plant natural products, 1-Hydroxybaccatin I was identified as a suppressor of the MKKS3–TRIM29 interaction (Fig. 7a, b). Co-IP assays in HT29 and SW480 cells treated with 1-Hydroxybaccatin I confirmed this effect (Fig. 7c). Furthermore, 1-Hydroxybaccatin I was found to reduce oxaliplatin resistance in MKKS3-overexpressing CRC cells (Fig. 7e, f). Molecular docking suggested that 1-Hydroxybaccatin I binds TRIM29 with His397, Asn511 and Thr513 in the OmpH domain (critical for MKKS3–TRIM29 interaction) potentially mediating this binding (Fig. 7d). Next, the anticancer properties of 1-Hydroxybaccatin I were evaluated in animal models. MKKS3-overexpressing MC38 cells were subcutaneously injected into mice, which were then divided into eight groups: oxaliplatin; 1-Hydroxybaccatin I; the known TRIM24–MKKS3 interaction inhibitor SB240111 (ref.^31^); 1-Hydroxybaccatin I + oxaliplatin; SB240111 + oxaliplatin; 1-Hydroxybaccatin I + oxaliplatin + IFNAR1-Ab; 1-Hydroxybaccatin I + oxaliplatin + CD8α-Ab; vehicle control. Oxaliplatin alone had negligible effects, whereas both 1-Hydroxybaccatin I and SB240111 individually reduced tumour burden. Combinations of these two agents with oxaliplatin showed a stronger anticancer effect, as demonstrated by tumour growth curves and weights (Fig. 7g). Consistent with in vitro results, IFNAR1-Ab effectively counteracted the inhibitory effect of the combination of 1-Hydroxybaccatin I and oxaliplatin (Fig. 7g). Given that CAFs secrete cytokines and chemokines modulating T cell function^32,33^, CD8α-Ab similarly abrogated this combination effect (Fig. 7g). These findings were further supported by Ki-67 staining (Fig. 7o). The therapeutic regimen showed a satisfactory safety profile, with no significant changes in body weight or haematological parameters (WBC, RBC, HGB, ALT, AST, PLT, %Lymphocyte; Supplementary Fig. 8a) and no notable histopathological abnormalities in organs (liver, kidney and lung) (Supplementary Fig. 8b). To further elucidate the role of 1-Hydroxybaccatin I in the tumour immune microenvironment, scRNA-seq was performed on MC38 xenograft tumours, and six distinct cell populations were classified (Fig. 7h; Supplementary Table 12). Notably, the proportions of T cells were significantly elevated in 1-Hydroxybaccatin I-treated tumours (12.54%), compared with controls (2.67%), whereas the percentage of fibroblasts was reduced to 2.68% in 1-Hydroxybaccatin I-treated tumours, compared with 4.51% in controls (Fig. 7h, i). However, the proportions of other cell types, such as macrophages/monocytes, were not significantly altered. KEGG analysis indicated significant enrichment of pathways related to cytokine signalling in fibroblasts (Fig. 7j; Supplementary Table 13) and T cell differentiation and activation pathway in T cells (Fig. 7k; Supplementary Table 14). SB24011-treated tumours were also analysed by scRNA-seq, and the results showed that the proportions of T cells (21.6%) were significantly elevated, compared with controls (Supplementary Fig. 9a). Effector function of iCAF was strengthened, as demonstrated by elevated expression of Il6, Cxcl1 and Cxcl2. Furthermore, myCAF markers (Acta2 and Tagln) were reduced both in the 1-Hydroxybaccatin I- and in the SB24011-treated groups (Fig. 7l; Supplementary Fig. 9b). Additionally, the effector function of T cells was strengthened, as shown by elevated expression of CD8a, Cd69 and Gzmb. Moreover, exhaustion markers including Pdcd1 were reduced both in the 1-Hydroxybaccatin I- and in the SB24011-treated groups (Fig. 7m; Supplementary Fig. 9c). These findings demonstrate that 1-Hydroxybaccatin I and SB24011 profoundly suppressed the iCAF-to-myCAF conversion and enhanced T cell function, suggesting a more potent antitumour response, which was consistent with the in vitro results. Flow cytometry of spleen and tumour lysates revealed that 1-Hydroxybaccatin I and SB240111 enhanced CD8^+^ T cell activation (CD69) and infiltration into tumours (Fig. 7n; Supplementary Fig. 9e, f), with further potentiation observed in the oxaliplatin combination group. In contrast, CD4^+^ T cells showed no significant alterations (Supplementary Fig. 9d). Additionally, GZMB was upregulated in the 1-Hydroxybaccatin I and SB240111 groups (Supplementary Fig. 9g). In sum, these results demonstrate that 1-Hydroxybaccatin I activates antitumour immunity, either alone or in combination with oxaliplatin, to inhibit CRC tumour progression without significant side effects in mice.

### Knockdown of MKKS3 enhances the sensitivity of colorectal cancer cells to oxaliplatin

**Fig. 8:**
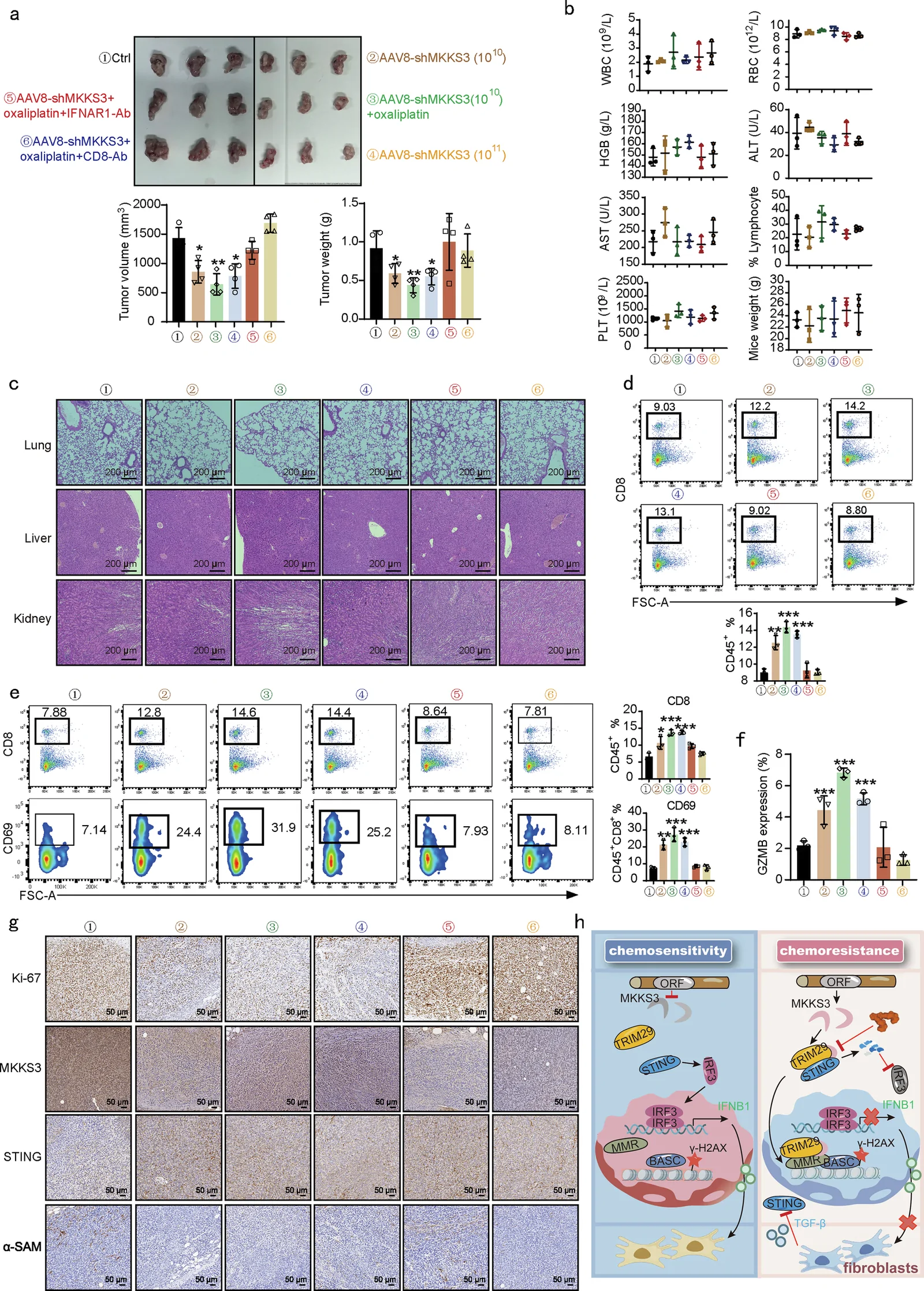
Knockdown of MKKS3 enhances the sensitivity of colorectal cancer cells to oxaliplatin. **a** Representative photographs of the MC38-bearing tumours from control (Ctrl) and treatment groups. AAV8-shMKKS3 (10^10^ vector genomes (vg) per mouse); AAV8-shMKKS3 (10^11^ vg per mouse); AAV8-shMKKS3 (10^10^ vg per mouse) + oxaliplatin (5 mg/kg); AAV8-shMKKS3 (10^10^ vg per mouse) + oxaliplatin (5 mg/kg) + IFNAR1-Ab (100 μg per mouse); AAV8-shMKKS3 (10^10^ vg per mouse) + oxaliplatin (5 mg/kg) + CD8α-Ab (200 μg per mouse). Tumour volume and weight of the eight groups were compared (low). **b** No significant differences were detected in the body weight of the mice or blood chemistry among the groups. WBC, white blood cells; RBC, red blood cells; HGB, haemoglobin; ALT, alanine aminotransferase; AST, aspartate aminotransferase; PLT, platelet. **c** Representative images of haematoxylin and eosin staining of the livers, kidneys and lungs; scale bar, 200 μm. **d**, **e** Flow cytometric analysis of CD8^+^ T cells in spleen (**d**) and tumour lysates (**e**): activation (CD69^+^) and infiltration of CD8^+^ T cells are enhanced by AAV8-MKKS3 treatment, with further potentiation when combined with oxaliplatin. **f** The tumour cells were stained with anti-CD8a and GRZB for flow-cytometry analysis. Statistics of GRZB expression. **g** Ki-67, MKKS3, STING and α-SAM staining of tumour sections. Scale bar, 50 μm. **h** Translationally regulated by DENR, MKKS3 directly interacts with TRIM29 to facilitate DNA repair complex assembly and induces STING ubiquitination and degradation, thus suppressing IFNβ secretion and activating fibroblasts. Activated fibroblasts secrete large amounts of TGFβ, which further downregulates STING expression and accelerates CRC progression. ORF, open reading frame. Bars, standard deviation; *P < 0.05; **P < 0.01; ***P < 0.001; ns, not significant.

To assess the in vivo therapeutic efficacy of MKKS3 knockdown (shMKKS3) in combination with oxaliplatin, MC38 cell-derived tumour xenografts were established. As shown in Fig. 8a, treatment with adeno-associated virus 8 (AAV8) targeting MKKS3 (AAV8-shMKKS3, 1 × 10^10^ vg/mice or 1 × 10^11^ vg/mice) resulted in a reduction in tumour burden, demonstrated by decreased tumour volume and weight. The combination of AAV8-shMKKS3 and oxaliplatin produced significantly enhanced therapeutic effects, including substantial reductions in tumour mass (Fig. 8a). However, the addition of IFNAR1-Ab and CD8α-Ab abolished the inhibitory effect of the AAV8-shMKKS3 and oxaliplatin combination (Fig. 8a). Furthermore, Ki-67 staining analysis revealed a significant reduction in cellular proliferation in the shMKKS3 and oxaliplatin group compared with other treatments, a phenomenon that was reversed by IFNAR1-Ab and CD8α-Ab treatment (Fig. 8a). The therapeutic regimen exhibited a satisfactory safety profile, with no substantial alterations in body weight or haematological parameters (WBC, RBC, HGB, ALT, AST, PLT, %Lymphocyte; Fig. 8b), and no significant histopathological abnormalities in the liver, kidney or lung (Fig. 8c). Flow cytometry of spleen and tumour lysates revealed that AAV8-shMKKS3 treatment enhanced CD8^+^ T cell activation (CD69) and tumour infiltration (Fig. 8d, e), with further potentiation observed when combined with oxaliplatin. Additionally, GZMB expression was upregulated in the AAV8-shMKKS3 or AAV8-shMKKS3 + oxaliplatin groups (Fig. 8f). IHC assays showed that the AAV8-shMKKS3 group exhibited increased STING expression and reduced MKKS3/α-SMA expression, consistent with the in vitro findings (Fig. 8g). Collectively, these results suggest that AAV8-shMKKS3, either alone or in combination with oxaliplatin, activates antitumour immunity and inhibits CRC tumour progression, without notable side effects, in mice.

## Discussion

The centrosomal shuttling protein MKKS, encoded by transcript variant 1, shares sequence homology with type II chaperonin family members, contributes to cytokinesis and interacts with other chaperonins to form the BBSome complex^34^. In this study, transcript variant 3 NR_072977, previously classified as a non-coding RNA (NR_072977) owing to the absence of key coding regions, was identified to encode microprotein MKKS3, which functions as a novel molecule in the regulation of tumour chemoresistance. Translationally regulated by DENR, MKKS3 can be expressed widespread across various human cancers. In CRC patients undergoing chemotherapy, high expression of MKKS3 correlates with poorer prognosis because MKKS3 diminishes oxaliplatin-induced DNA damage and activates fibroblasts (CAFs) to promote chemoresistance. By interacting with TRIM29, MKKS3 facilitates DNA repair complex assembly and induces STING ubiquitination and degradation, thus suppressing IFNβ secretion and activating fibroblasts (Fig. 8h). Activated CAFs can secrete large amounts of TGFβ, which further downregulates STING expression and accelerates CRC progression.

The tumour microenvironment (TME) constitutes a complex ecological niche encompassing non-tumour cells, stromal elements and the repertoire of molecules that they secrete. Dynamic and persistent interactions between cancer cells and their surrounding TME substantially influence tumour initiation, progression, dissemination and the efficacy of therapeutic interventions^35^. Cancer cells can achieve drug tolerance via sophisticated cell–cell communication networks. Both refractory cells and TME constituents represent key drivers of drug resistance, communicating with chemosensitive cells through extracellular vesicles or various soluble mediators including cytokines and chemokines^36^. CAFs play a pivotal role in the formation of the TME, which typically exhibits immunosuppressive properties^37^. Under normal physiological conditions, immune cells such as T cells, NK cells and macrophages are capable of eliminating tumour cells, thereby regulating and inhibiting tumour growth. CAFs influence T cell phenotypic changes, activate NK cell functions, and polarize macrophages towards the M2 phenotype by secreting cytokines and chemokines or through indirect interactions with immune cells within the TME, thus contributing to immune suppression and tumour progression^38–41^. Gene-expression analyses from patients undergoing immune-checkpoint blockade also revealed a correlation between elevated CAF levels and poor responses to immunotherapy, suggesting that targeting CAFs could alleviate cancer drug resistance^42^. In this study, we found that MKKS3 knockdown suppressed the activation of fibroblasts, enhanced CD8^+^ T cell activation and tumour infiltration. Furthermore, the expression of GZMB, a key effector molecule of cytotoxic T cells, was significantly elevated upon MKKS3 knockdown (Fig. 7). These results indicate that MKKS3 regulates CD8^+^ T cell function by modulating fibroblast activation. The combination of MKKS3-targeted therapy with oxaliplatin-based chemotherapy or immune-checkpoint inhibitors may represent a promising therapeutic approach to overcome chemoresistance and improve clinical outcomes in patients with CRC.

1-Hydroxybaccatin I, a natural diterpenoid derived from *Taxus* species and a crucial precursor in the semi-synthesis of paclitaxel^43^, has been infrequently studied in the context of CRC. Our research indicates that this compound inhibits the interaction between TRIM29 and MKKS3. By directly binding to TRIM29 in the MKKS3–TRIM29 interaction, 1-Hydroxybaccatin I interrupts the signalling pathway of MKKS3 through TRIM29 to suppress tumour proliferation. This tumour suppression effect can be further enhanced in its combination treatment with oxaliplatin.

Oxaliplatin-based regimens represent the standard first-line therapy for advanced or metastatic CRC. However, chemoresistance remains a major clinical challenge. lncRNA-encoded microproteins significantly expand the universe of potential drug targets for cancers, where certain microproteins play critical roles. This study demonstrated that MKKS3 suppression significantly enhanced oxaliplatin sensitivity, presenting the therapeutic potential of combining MKKS3 inhibition by 1-Hydroxybaccatin I or MKKS3 knockdown with oxaliplatin in overcoming chemoresistant CRC. Our findings highlight the clinical translational value of MKKS3 inhibition as a promising therapeutic strategy for CRC, particularly for patients with high fibroblast activation. 1-Hydroxybaccatin I, an inhibitor of MKKS3, can therefore be further explored to be a potential anti-CRC drug synergistic with oxaliplatin to combat the chemotherapy resistance mediated by MKKS3. Collectively, our study provides a feasible research strategy for addressing the therapeutic challenges of targeting non-coding RNAs by focusing on their encoded and functionally characterized microproteins.

In conclusion, this study discovers that NR_072977 can encode the microprotein MKKS3 to bind with TRIM29, promoting the assembly of DNA-repair complexes. Through this interaction of MKKS3 with TRIM29, MKKS3 induces STING ubiquitination and degradation, suppressing IFNβ secretion. The reduction in IFNβ enhances fibroblast activation, further decreasing STING expression and promoting CRC progression through TGFβ secretion. These findings once again validate our previous judgement that the hidden proteome expressed by “lncRNAs” may contain important functional molecules. This result also offers new insights into the molecular mechanisms underlying CRC oxaliplatin resistance, with new protein MKKS3 being a novel target in the crucial signalling pathway.

## Supplementary information

Supplementary Information

Supplementary Table 1-10

Supplementary Table 11

Supplementary Table 12

Supplementary Table 13

Supplementary Table 14

raw data 1

## Data Availability

The data from patients with CRC used in this study are available in the Gene Expression Omnibus Repository database, under the accession codes GSE72970 and GSE69657. These data are publicly available without access restrictions. Datasets for MKKS3 receiver-operating characteristic curves were obtained from the Clinical Proteomic Tumor Analysis Consortium (PDC000166). The single-cell RNA sequencing data were obtained from the Gene Expression Omnibus Repository (accession number GSE328757). The proteomics and CRISPR/Cas9 screening databases are available on figshare: 10.6084/m9.figshare.32054904 and crisper-10.6084/m9.figshare.32054145. The data of this study are available from the corresponding authors upon reasonable request.
