# Supplementary material for: The long non-coding RNA-encoded microprotein MKKS3 drives colorectal cancer chemoresistance
Source: Exp Mol Med. 2026 Sep 2;58(9):2919–37. doi: 10.1038/s12276-026-01817-w (PMC13633250; doi:10.1038/s12276-026-01817-w)
Supplement: Supplementary file 1 [file 12276_2026_1817_MOESM1_ESM.pdf]

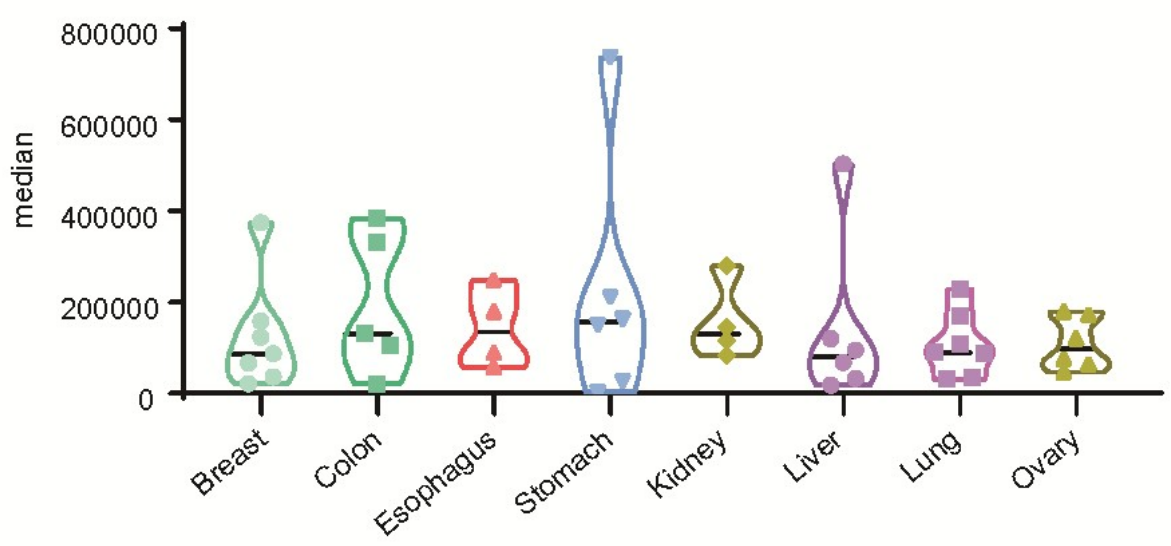

Supplementary Fig. 1: The absolute numbers of potential proteins identified in cancers

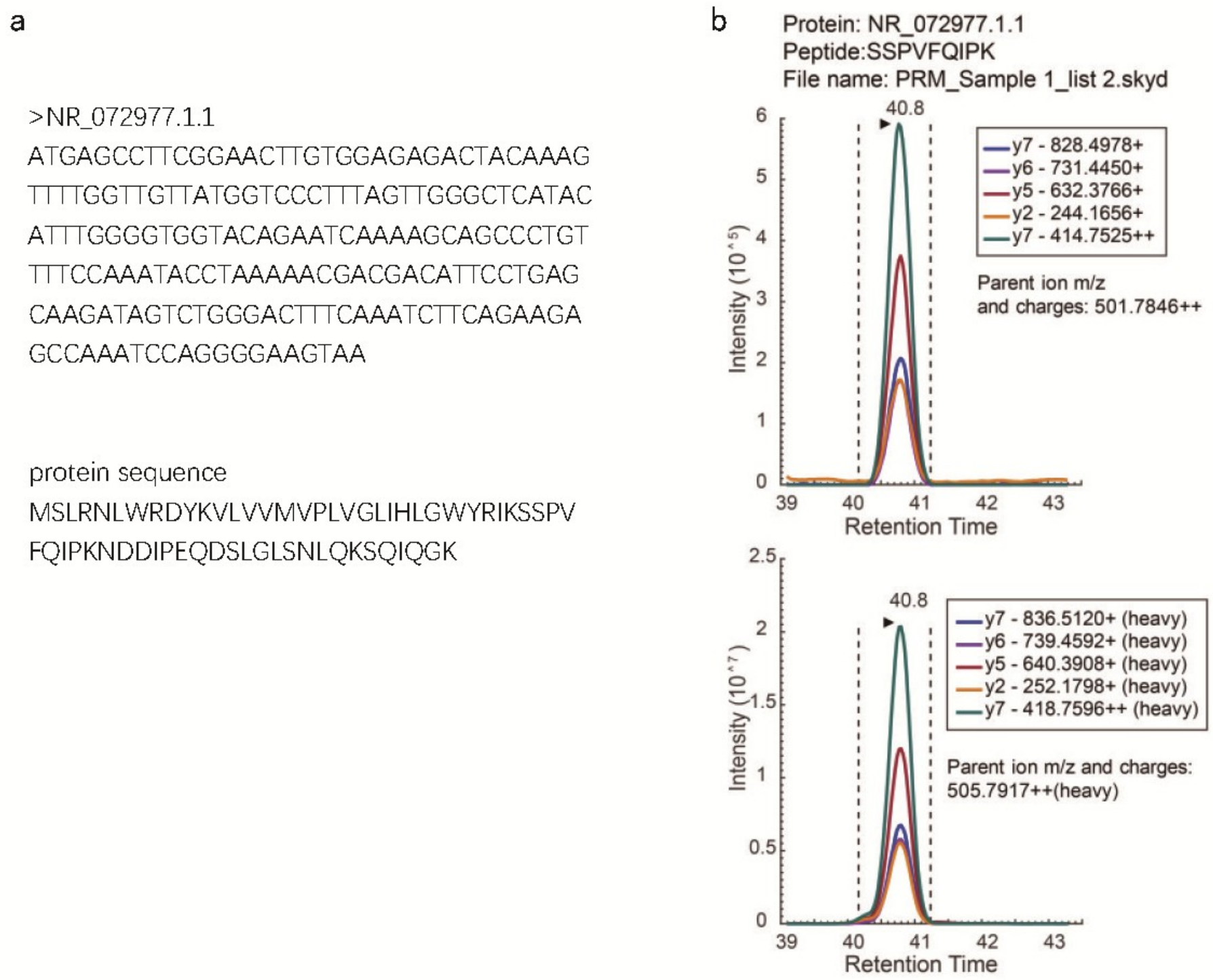

Supplementary Fig. 2: NR 072977 encoded the protein MKKS3

(a) Bioinformatics analysis indicated a 189-nucleotide ORF in NR 072977 with the potential to translate a 63-amino acid protein. (b) The peptide of the MKKS3 protein was identified by mass spectrometry.

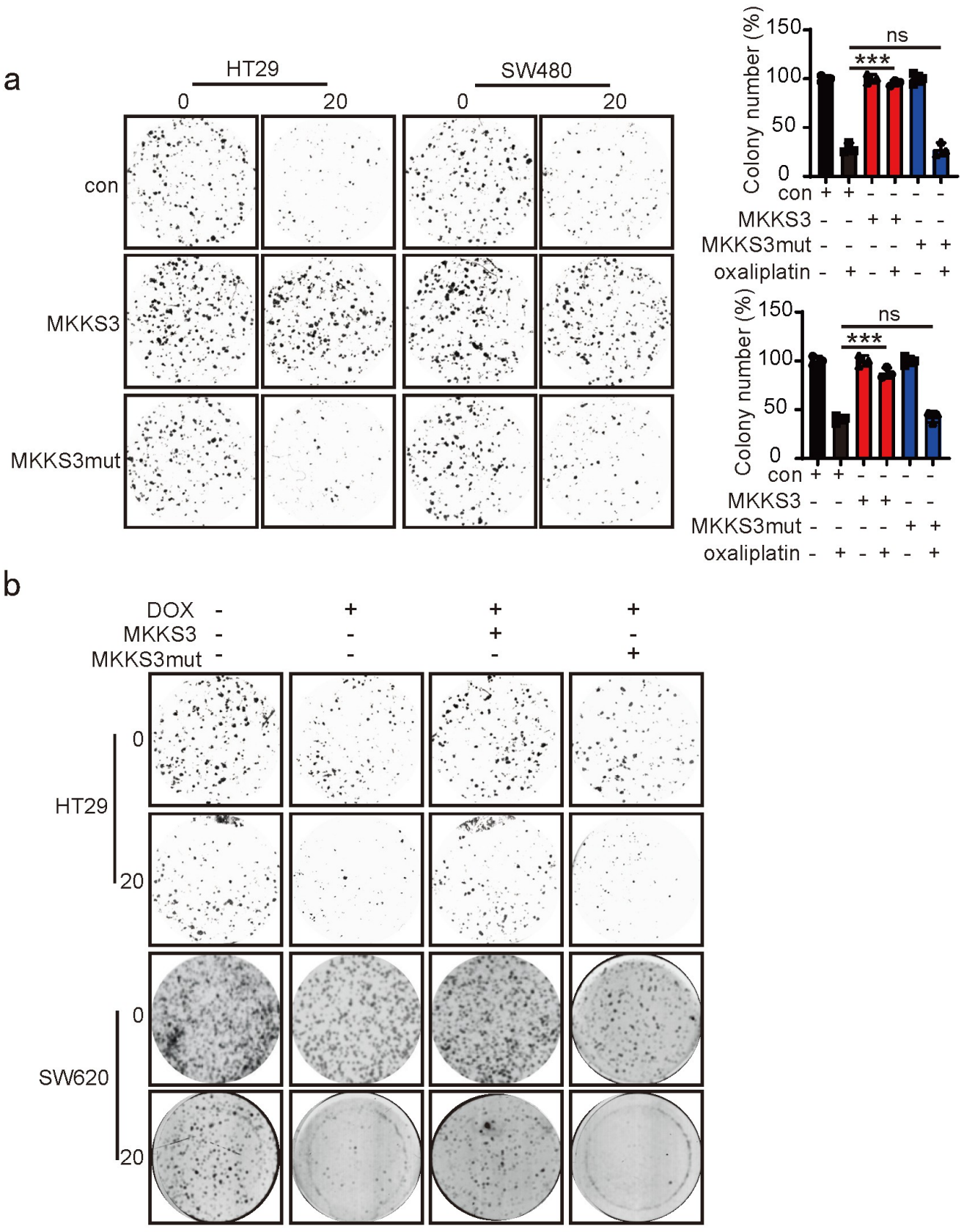

Supplementary Fig. 3: Functional validation of MKKS3 in CRC oxaliplatin resistance

(a) The HT29 and SW480 cells expressing MKKS3 or MKKS3mut were treated with oxaliplatin (20 μM) for 1-4 days, and the cell proliferation was analyzed by colony-formation assay. (b) Cells were treated with DOX (1 mg/mL) for 48 hours, then re-expressed MKKS3 or MKKS3mut, followed treated with oxaliplatin (20 μM) for 1-4 days. The oxaliplatin resistance was analyzed USING colony-formation assays.

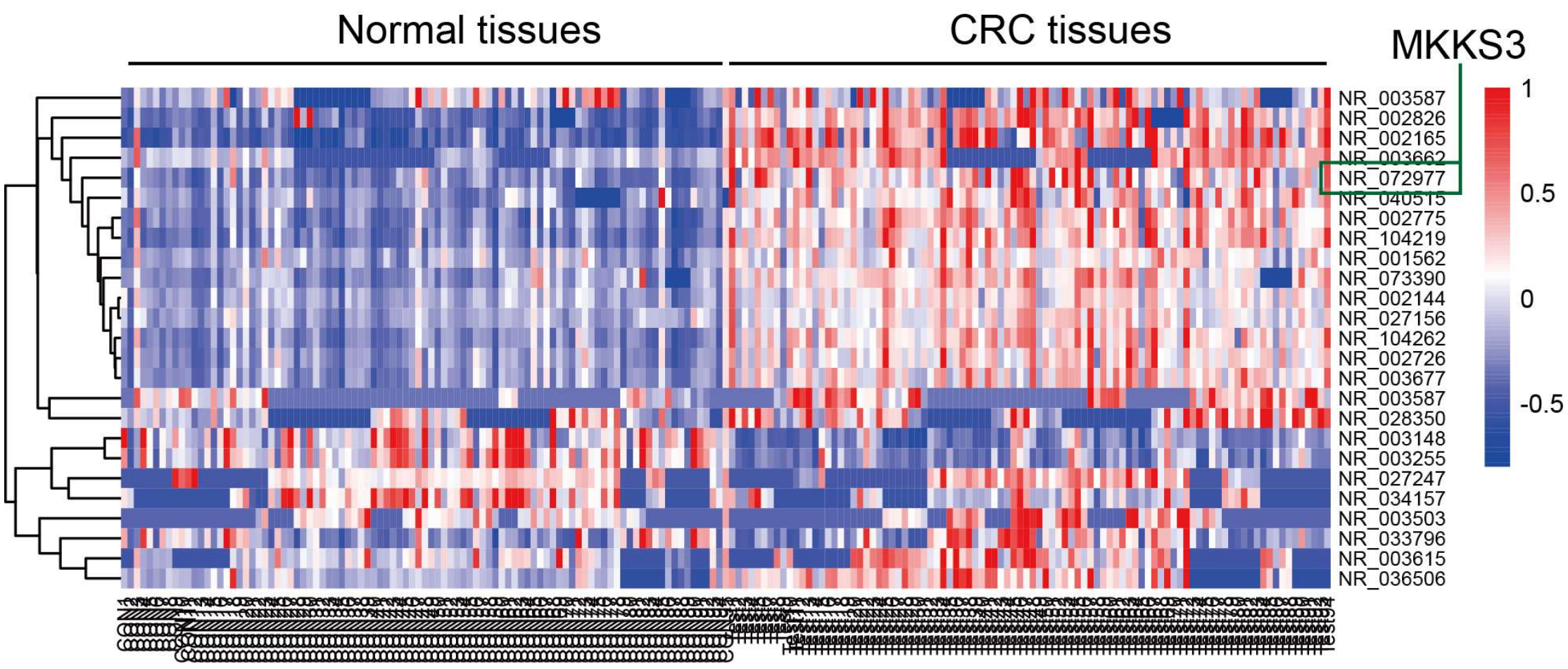

Supplementary Fig. 4: NR 072977-encoded MKKS3 expression level in normal and CRC

tissues

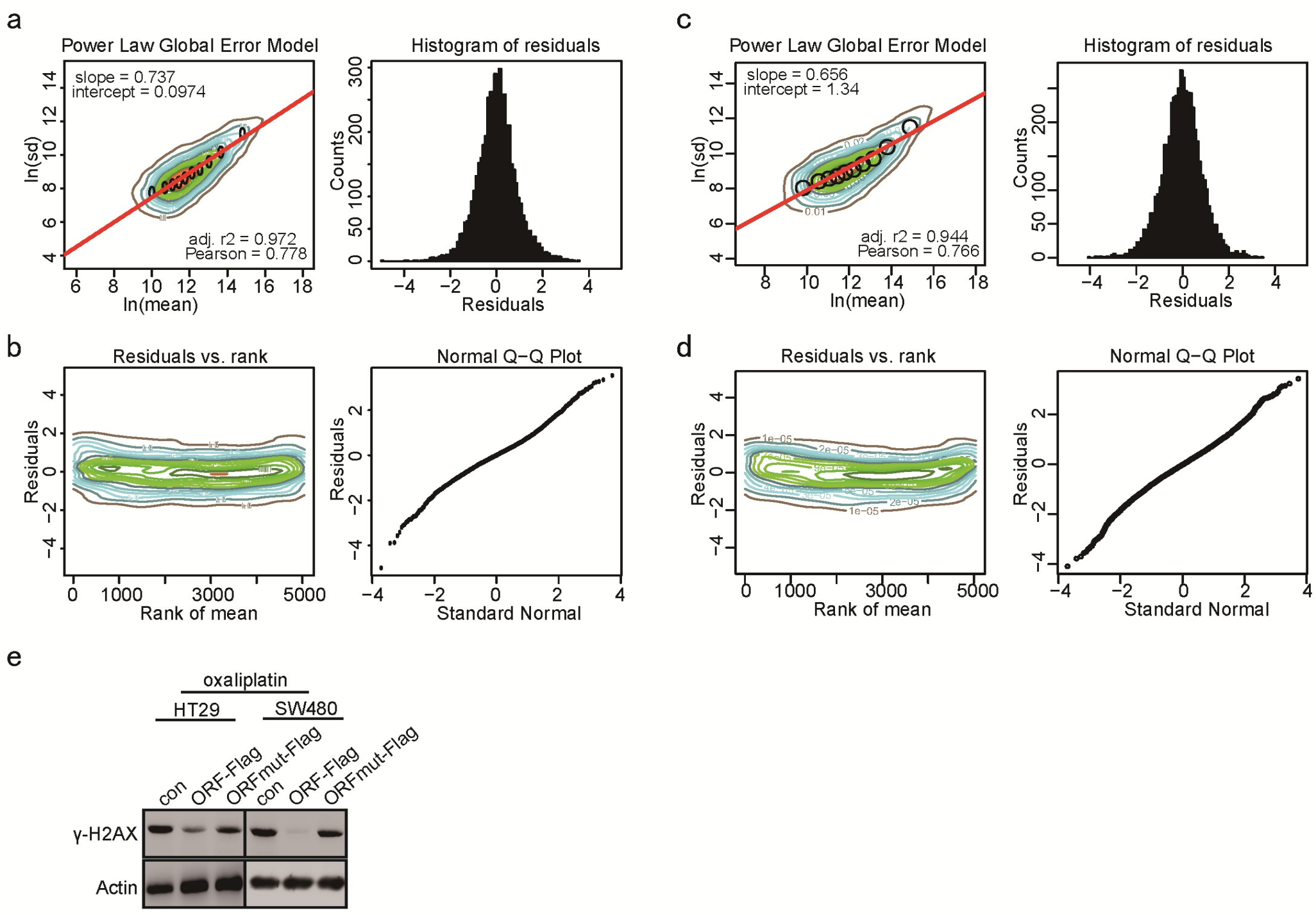

Supplementary Fig. 5: DIA-based proteomics data of CRC cells

(a-d) Quality control of DIA-based proteomics data: PLGEM algorithm analysis showing linear correlation of mean and SD of normalized protein quantitation values (correlation coefficients = 0.972 for HT29, 0.944 for SW480) (a, c); Q-Q plots demonstrating normal distribution of residuals, confirming good data reproducibility (b, d). (e) MKKS3-overexpressing, MKKS3mut-overexpressing, and control cells were treated with oxaliplatin (20 µM, 48 h). The expression of γ-H2AX was analyzed by western blotting.

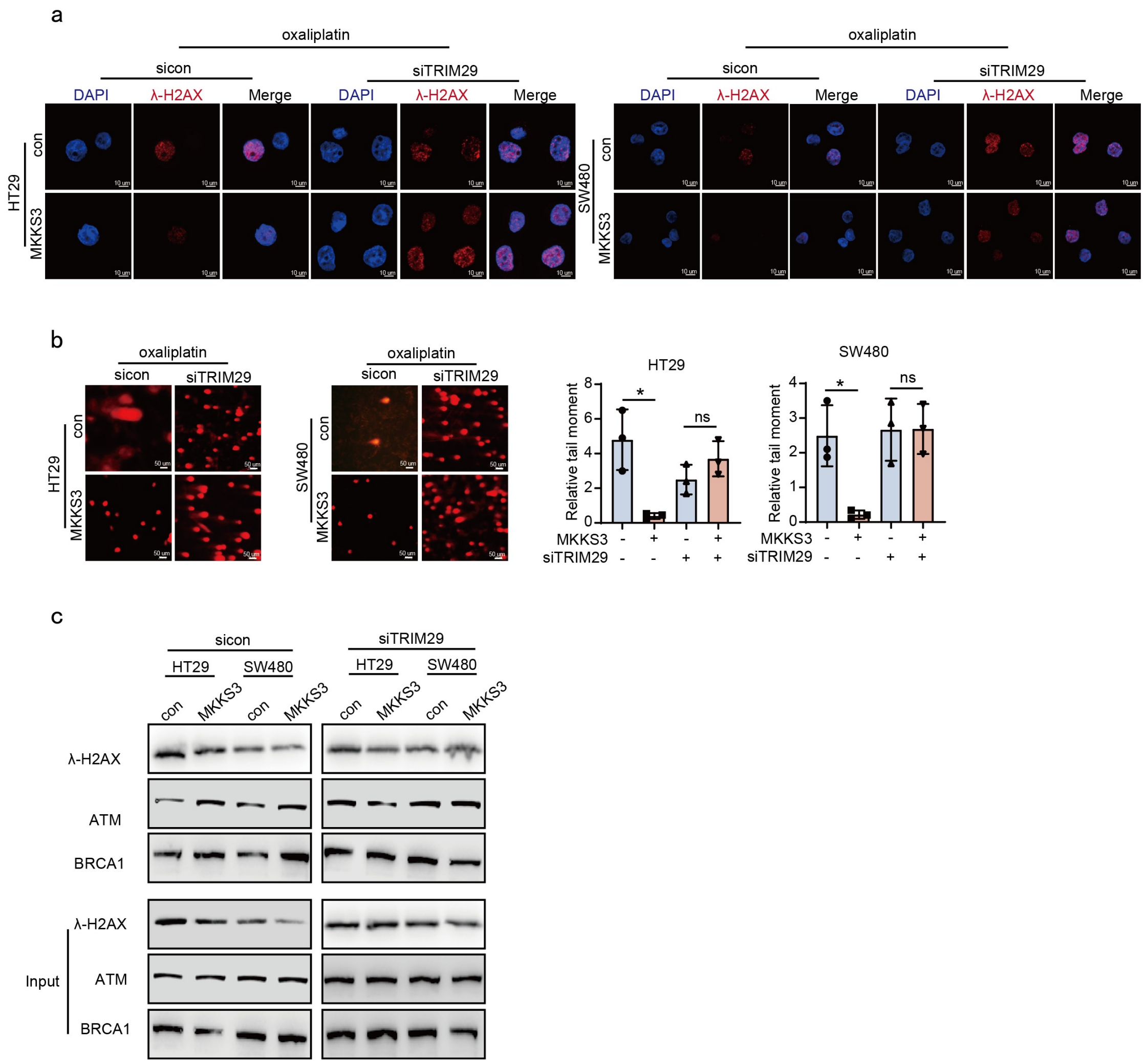

Supplementary Fig. 6: DIA-based proteomics data of CRC cells

(a-b) In TRIM29-knockdown CRC cells, MKKS3 was overexpressed, and oxaliplatin-induced DNA damage was evaluated using the comet assay (b, Scale bar, 10 μm) and γ-H2AX immunofluorescence staining (a, Scale bar, 50 μm). Blue, DAPI; red, γ-H2AX. (c) Co-IP assay demonstrated that TRIM29 knockdown in CRC cells completely abolished the effect induced by MKKS3 overexpression on the interaction among ATM, γ-H2AX, and BRCA1. Bars, SD; *p < 0.05; **p < 0.01; ***p < 0.001; ns, nonsignificant.

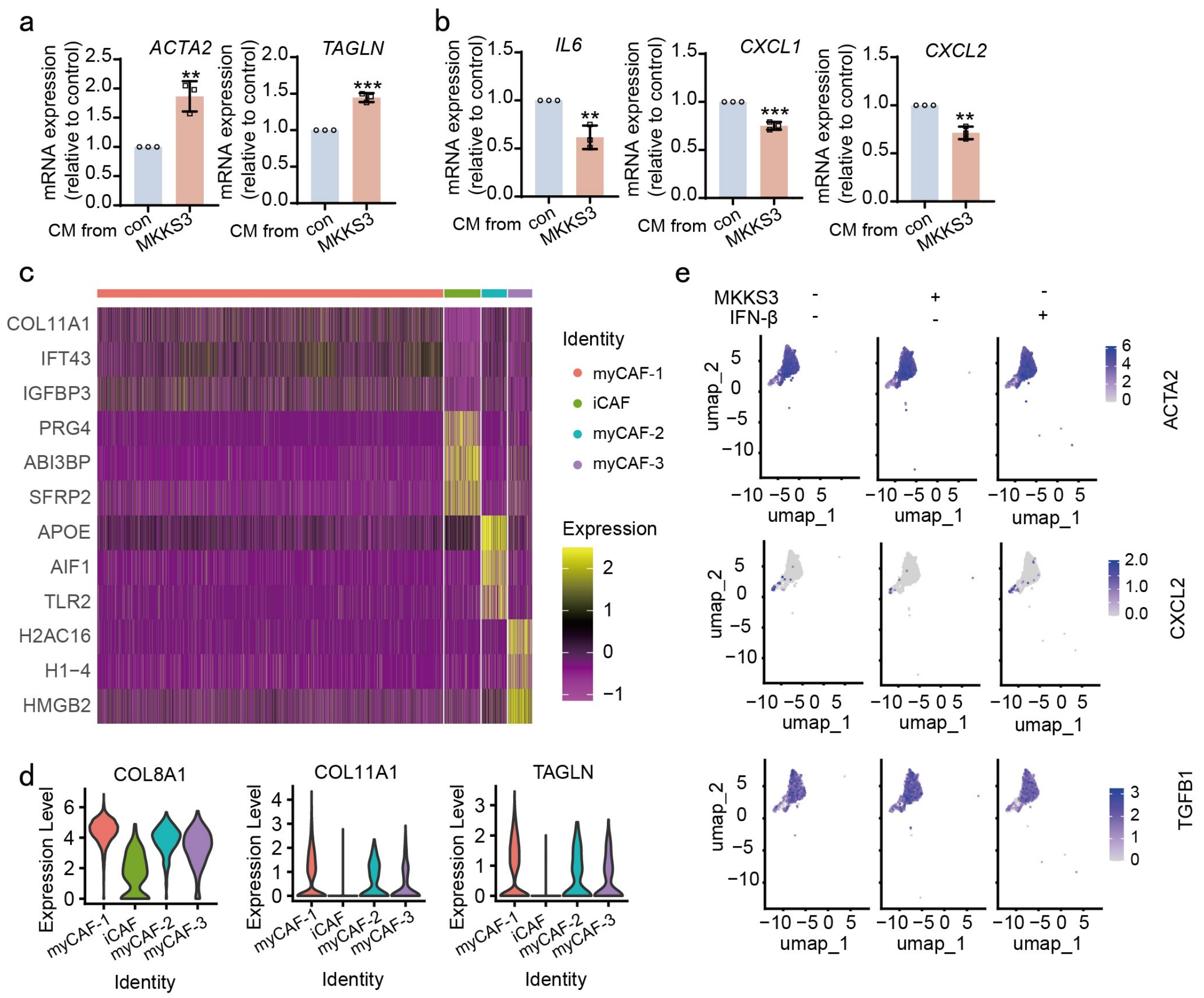

Supplementary Fig. 7: MKKS3 drove iCAF-to-myCAF conversion

(a-b) The mRNA expression of iCAF and myCAF-related markers was analyzed by qPCR. (c-d) Three of these clusters consistently showed high expression of myCAFs markers, including COL8A1, COL11A1, and TAGLN, and were collectively annotated as myCAF subtypes. (e) Expression of myCAF markers, iCAF markers, and TGFB1 of fibroblasts. Bars, SD; *p < 0.05; **p < 0.01; ***p < 0.001; ns, nonsignificant.

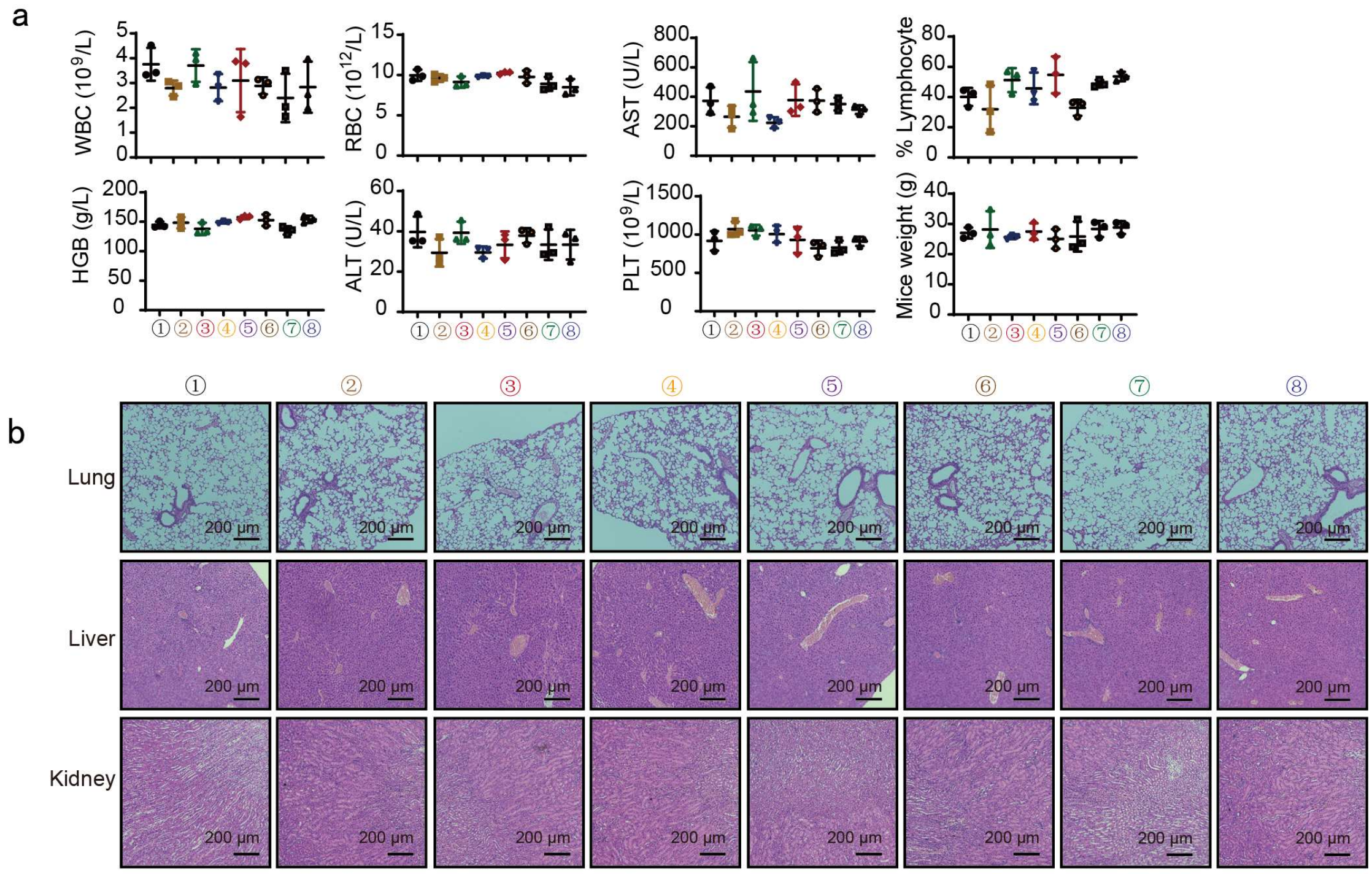

Supplementary Fig. 8: 1-Hydroxybaccatin I exerts antitumor effects without inducing additional toxicity in CRC xenograft models

(a) No significant differences were detected in body weight or blood chemistry among the groups; WBC, white blood cells; RBC, red blood cells; HGB, hemoglobin; ALT, alanine aminotransferase; AST, aspartate aminotransferase; PLT, platelet; and %Lymphocyte. (b) Representative images of H&E staining of the livers, kidneys, and lungs; scale bar, 200 μm. Bars, SD; $*p < 0.05$; $**p < 0.01$; $***p < 0.001$; ns, nonsignificant.

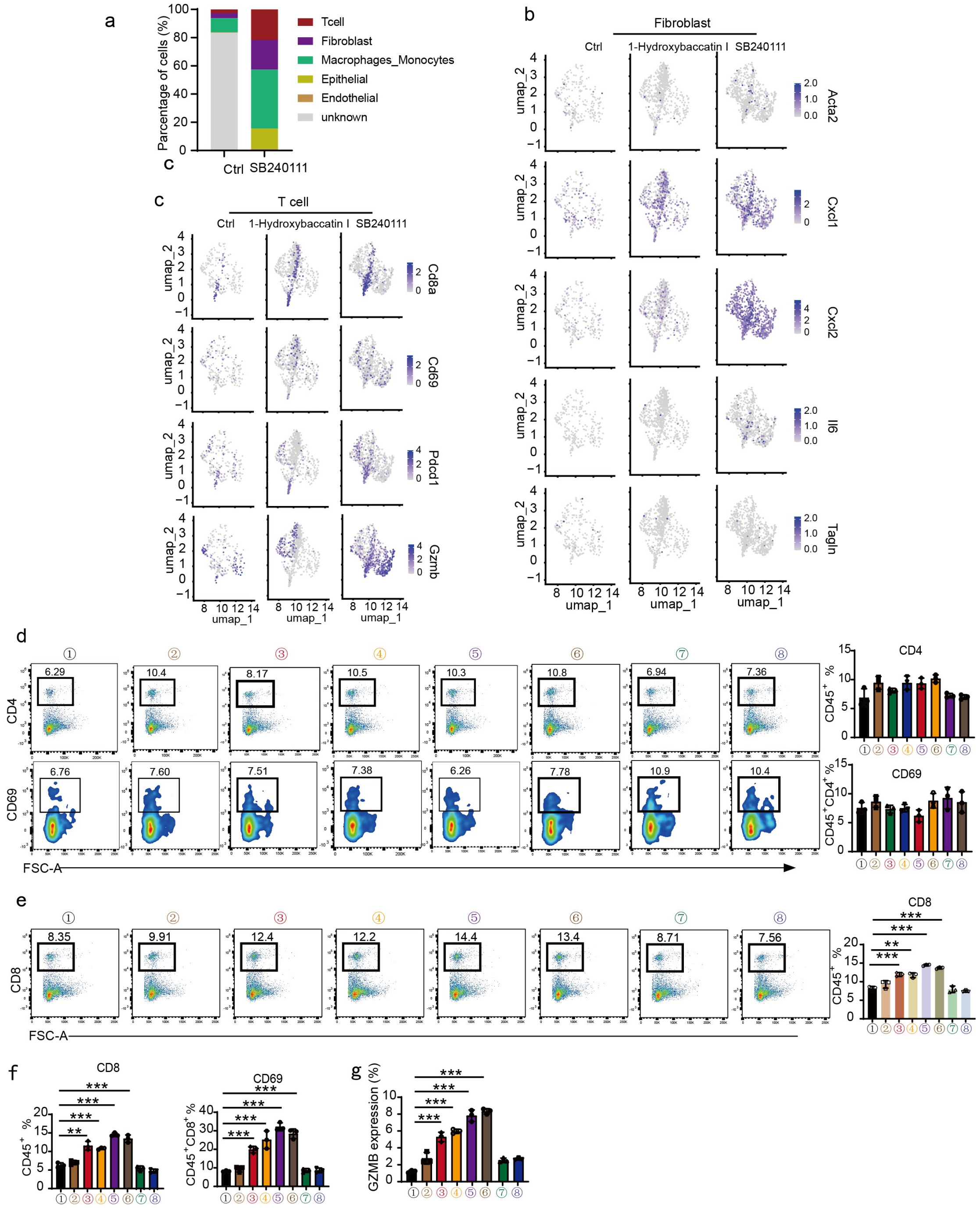

Supplementary Fig. 9: 1-Hydroxybaccatin I profoundly enhanced T cell function

(a) Proportional distribution of cell types. (b) Expression of myCAF markers (Tagln, Acta2) and iCAF markers (Il6, Cxcl1, Cxcl2) of fibroblasts. (c) Expression of Gzmb, Pdcd1, Cd69, and Cd8a of T cells. (d) Flow cytometric analysis of $CD4^+$ T cells in tumor lysates. (e) Flow cytometry analysis of $CD8^+$ T cells in spleen. (f) Activation ($CD69^+$) and infiltration of $CD8^+$ T cells were enhanced by 1-Hydroxybaccatin I or SB240111 treatment, with further potentiation when combined with

oxaliplatin. (g) Tumor cells stained with anti-CD8a and GZMB for flow cytometry analysis. Statistics of GZMB expression are shown. Bars, SD; *$p < 0.05$; **$p < 0.01$; ***$p < 0.001$; ns, nonsignificant.
