# Supplementary material for: The long non-coding RNA-encoded microprotein MKKS3 drives colorectal cancer chemoresistance
Source: Exp Mol Med. 2026 Sep 2;58(9):2919–37. doi: 10.1038/s12276-026-01817-w (PMC13633250; doi:10.1038/s12276-026-01817-w)
Supplement: Supplementary file 2 [file 12276_2026_1817_MOESM7_ESM.pdf]

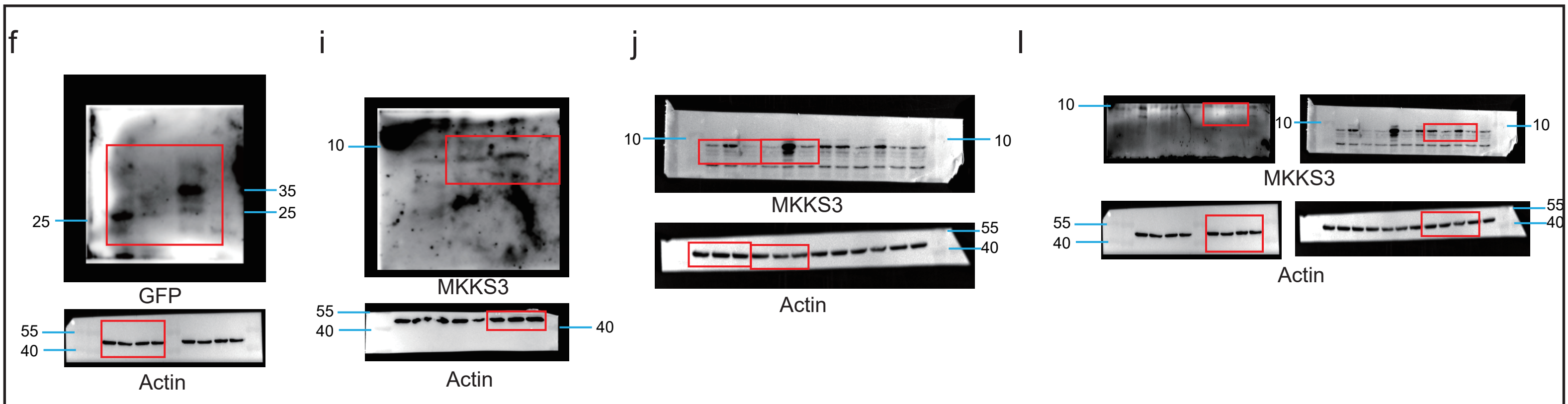

Figure 2

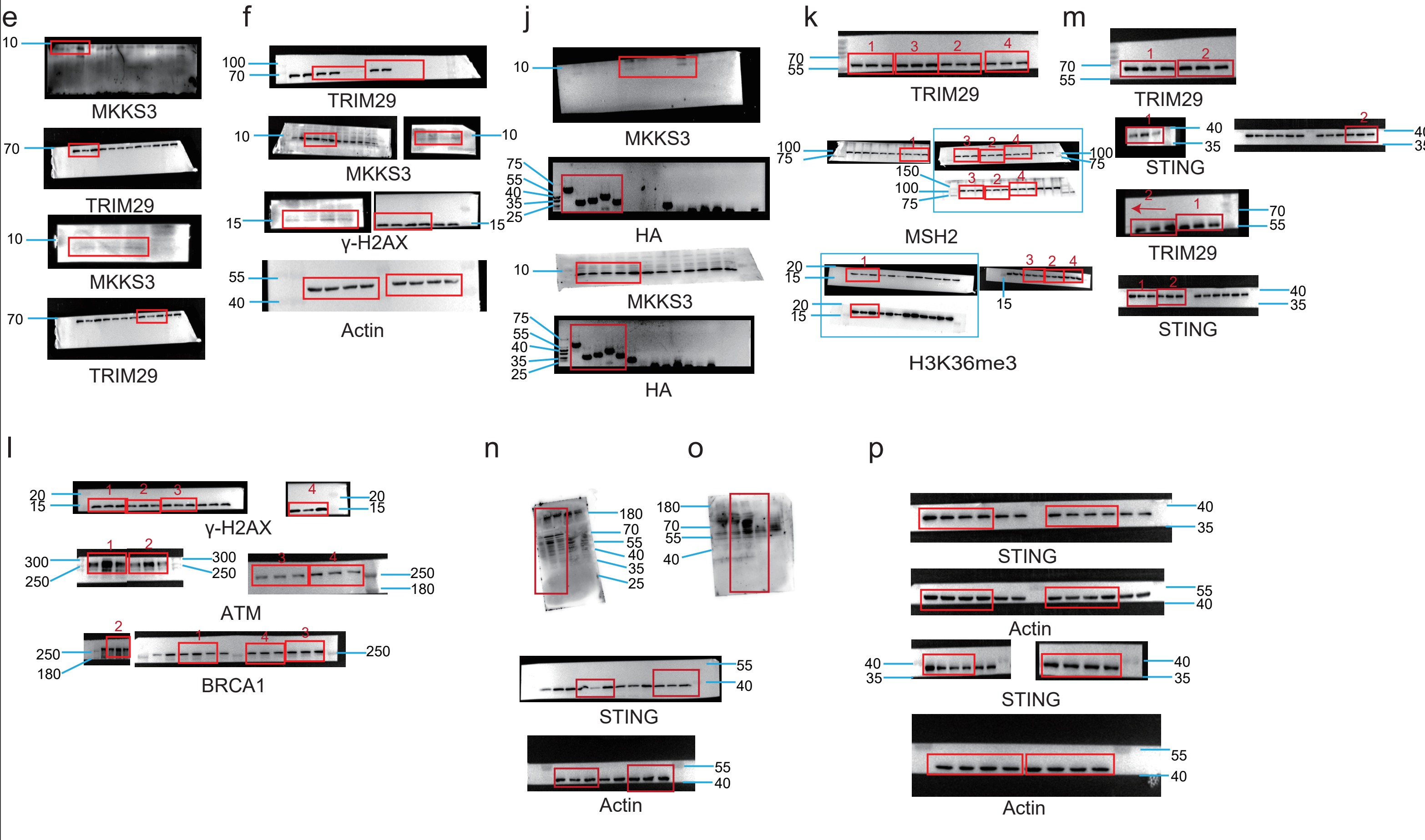

Figure 5

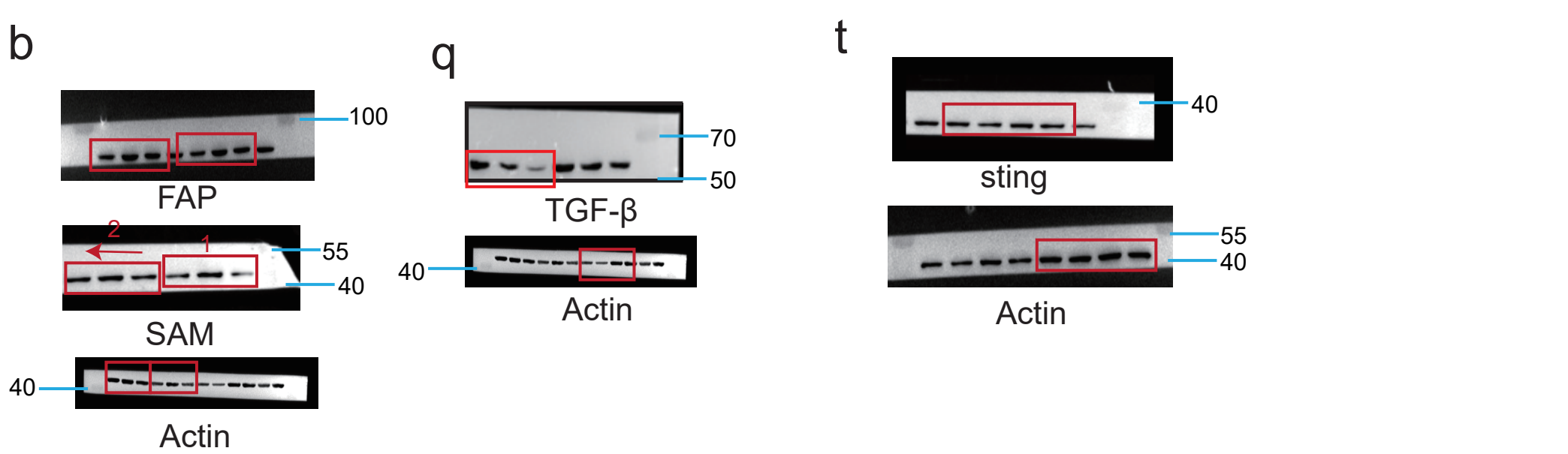

Figure 6

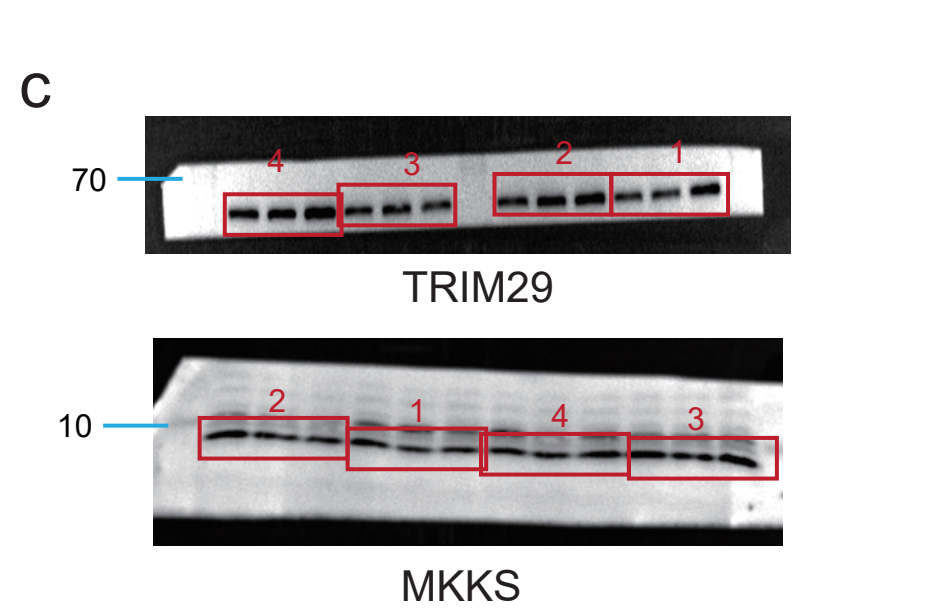

Figure 7

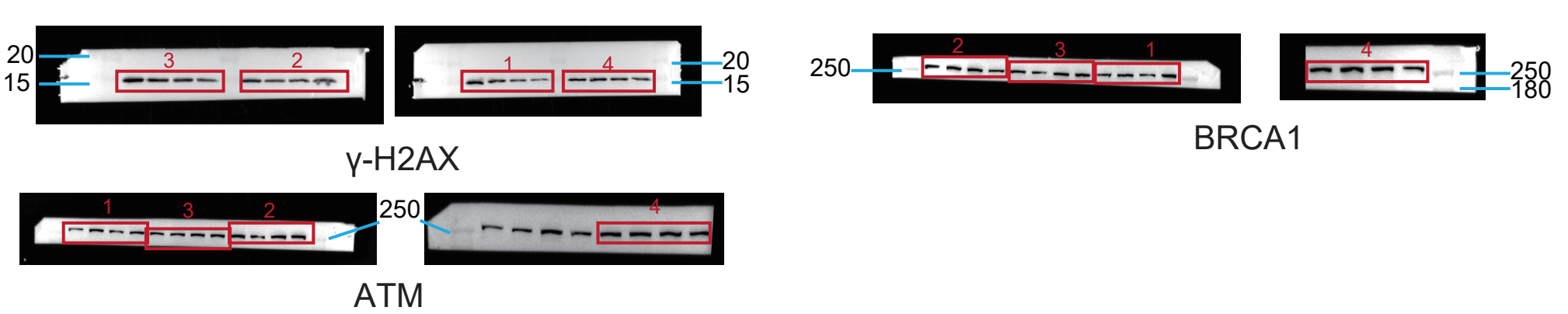

Figure S6
